# Reciprocal transport of carbon and nitrogen between plants and the hyphosphere microbiome in a temperate grassland

**DOI:** 10.1111/nph.71576

**Published:** 2026-09-10

**Authors:** Luise Brandt, Anna Abrahão, Ann‐Christin Wicht, Stefanie Schulz, Michael Schloter, Steffen Schweizer, Sven Marhan, Ellen Kandeler

**Affiliations:** ^1^ Department of Soil Biology Institute of Soil Science and Land Evaluation, University of Hohenheim Emil‐Wolff‐Straße 27 Stuttgart 70599 Germany; ^2^ Department of Ecology Universidade de Brasília Campus Universitário Darcy Ribeiro Brasília 70910‐900 Brazil; ^3^ Research Unit Comparative Microbiome Analysis Helmholtz Zentrum Munich Ingolstädter Landstraße 1 Neuherberg 85764 Germany; ^4^ Chair of Environmental Microbiology, TUM School of Life Sciences Technical University of Munich Emil‐Ramann‐Straße 2 Freising 85354 Germany; ^5^ Chair of Soil Science, TUM School of Life Sciences Technical University of Munich Emil‐Ramann‐Straße 2 Freising 85354 Germany

**Keywords:** arbuscular mycorrhizal fungi, carbon–nitrogen exchange, cross‐kingdom microbial networks, fungal–bacterial interactions, grassland soil, hyphosphere, NanoSIMS, stable isotope labeling

## Abstract

- Despite its relevance for plant nutrient acquisition, the role of the hyphosphere in structuring fungal–bacterial networks and mediating carbon–nitrogen (C–N) exchange under field conditions remains unclear. We therefore examined reciprocal C–N fluxes along the plant–fungi–bacteria continuum and their link to microbial community assembly across grassland soil microhabitats.
- Sterilized compartmentalized ingrowth cores were exposed to soil in the field for 3 months. Plants were pulse‐labeled with ^13^CO_2_ and ^13^C was traced into microbial biomarkers over 7 d. ^15^N‐labeled urea was supplied to a hyphal compartment and isotope recovery in plants was quantified. We reconstructed microbial networks and visualized microscale isotope distributions.
- Plant‐derived ^13^C was rapidly incorporated into fungi within 1 d and into hyphosphere bacteria after 7 d. Conversely, 16.9% of the initially added 150 μg ^15^N was transferred from the hyphal compartment to plants. Microbial diversity declined from the rooted toward the hyphal‐dominated compartments, while networks reorganized with relatively more positive fungal–bacterial associations.
- Arbuscular mycorrhizal fungi act as primary channels of reciprocal C–N exchange, rapidly transporting newly fixed plant C into root‐distant soil zones. More broadly, soil fungi act as ecological filters shaping bacterial assembly and may foster increasingly positive associations with bacteria in hyphal‐dominated habitats.

## Graphical abstract

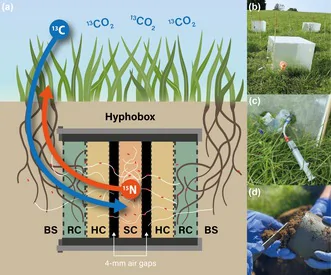

## Introduction

A substantial fraction of fixed photosynthates in grasslands is quickly allocated belowground (Pausch & Kuzyakov, 2018), fueling soil microbial activity, microbial nutrient mobilization, and nutrient uptake by plants (Jones *et al*., 2004). This reciprocal exchange of carbon (C) and nutrients between plants and soil microbes takes place within a highly heterogeneous soil matrix structured by spatially distinct microhabitats. Among these, the rhizosphere, the soil zone influenced by plant roots, and hyphosphere, the soil zone influenced by fungal hyphae, constitute particularly important interfaces for the C and nutrient exchange among organisms of different trophic levels (Duan *et al*., 2025). Yet, while rhizosphere processes have been intensively studied, the dynamics of C and nutrient flow and microbial interactions in the root‐free hyphosphere remain less resolved, particularly under field conditions.

In exchange for photosynthetically fixed C (on average 6.2% of a host plant's net primary production), arbuscular mycorrhizal fungi (AMF) facilitate host plant acquisition of nutrients and water in grasslands (Hawkins *et al*., 2023). Plant‐derived C is transferred to AMF within hours (Johnson *et al*., 2002), where it is respired, incorporated into fungal biomass, or released into soil via exudation and hyphal turnover (Toljander *et al*., 2007; Jones *et al*., 2009; Kramer *et al*., 2012). Beyond plant–fungal C exchange in mycorrhizal symbioses, saprotrophic fungi can rapidly assimilate plant‐derived C from rhizodeposition (Huang *et al*., 2020), thereby further contributing to the redistribution of newly fixed C in soil. These two fungal guilds differ fundamentally in how and where they access plant‐derived C: AMF receive photosynthates directly from living host roots via intracellular symbiotic structures, whereas saprotrophic fungi largely acquire C through extracellular enzymatic decomposition of dead plant material. Despite these contrasting nutritional strategies, both form extensive hyphal networks that bridge soil pores and create a unique microhabitat associated with their hyphae. The hyphosphere is characterized by hyphal exudation and turnover and provides surfaces that promote bacterial attachment and dispersal (Toljander *et al*., 2006, 2007; Lecomte *et al*., 2011; Kaiser *et al*., 2015; See *et al*., 2022), thereby shaping intense fungal–bacterial interactions. Notably, AMF lack the enzymatic capacity to independently degrade complex organic N and P sources and therefore largely rely on their hyphosphere microbiome to mineralize these nutrients before uptake (Rozmoš *et al*., 2022; Duan *et al*., 2024; Vaishnav *et al*., 2025). Consequently, a tripartite plant–fungal–bacterial continuum is established, in which fungi channel plant photosynthates to hyphosphere microbes and, in the case of AMF, return microbially mobilized nutrients from the hyphosphere to plants. However, while isotope‐labeling studies have demonstrated that plant‐derived C can reach hyphosphere bacteria, and ultimately, stable soil organic matter (OM) pools over weeks to months (Kakouridis *et al*., 2024), the initial fate of newly fixed C and its partitioning among fungal and bacterial groups remain poorly resolved. Moreover, demonstrating reciprocal nutrient fluxes from root‐free nutrient hotspots back to plants requires experimental approaches that disentangle hyphal transport from diffusion through soil solution and direct root uptake.

Beyond elemental fluxes, fungal–bacterial interactions are embedded within complex microbial networks that may vary across soil microhabitats and range from facilitative to competitive. Regarding positive interactions, bacteria may benefit from fungal exudates (Toljander *et al*., 2006, 2007) and hyphal‐mediated transport pathways (Warmink *et al*., 2011; Otto *et al*., 2017), while fungi may profit from bacterial nutrient mobilization (Bukovská *et al*., 2018; Hestrin *et al*., 2019; Rozmoš *et al*., 2022), protection from environmental stresses (Büttner *et al*., 2021; Sun *et al*., 2023), or mineral weathering (Frey‐Klett *et al*., 2007). Hyphosphere‐associated microbial communities harbor distinct bacterial assemblages, including Proteobacteria, Actinobacteria, and Bacteroidetes consistently reported as enriched core taxa, many of which are assumed to mediate nutrient cycling (Nuccio *et al*., 2013; Emmett *et al*., 2021; Wang *et al*., 2022). More recently, microbial co‐occurrence network analyses have revealed distinct interaction structures within these hyphosphere communities (Jin *et al*., 2024; Kakouridis *et al*., 2024; Slanzon *et al*., 2025). However, most existing studies on fungal–bacterial interactions in the hyphosphere have been conducted under controlled laboratory conditions, and field‐based evidence on the structure and dynamics of hyphosphere microbial networks in natural ecosystems remains limited (Yuan *et al*., 2021; Wang *et al*., 2024).

The aim of this study was to resolve the temporal dynamics of C and nitrogen (N) exchange between plants and hyphosphere microbes and to determine how this is related to microbial communities and fungal–bacterial co‐occurrence networks across root‐ and hyphae‐associated soil microhabitats in a temperate grassland. For this, we established compartmentalized ingrowth cores (‘hyphoboxes’) *in situ*, separating a root‐free hyphosphere zone from rooted soil. Hyphobox compartments were filled with sterilized soil, relying on microbial colonization from the surrounding intact soil. We combined ^13^CO_2_ pulse labeling of grassland plants with the addition of a ^15^N‐labeled OM hotspot located within the hyphosphere zone to investigate reciprocal C and N fluxes between plants and hyphosphere microbes. We traced ^13^C into microbial lipid biomarkers across a short‐term 7‐d period to identify fungal and bacterial groups actively incorporating newly plant‐fixed C, reconstructed cross‐kingdom microbial networks and used NanoSIMS to visualize small‐scale isotopic distributions in the different soil habitats.

We hypothesized that (1) plants and hyphosphere microbes engage in reciprocal C–N exchange mediated by fungal hyphal networks. Specifically, we expected newly fixed plant C to be rapidly transferred belowground and first incorporated by fungi, particularly AMF, followed by delayed incorporation into hyphosphere bacteria, consistent with indirect bacterial access to plant‐derived C via fungal exudation, turnover, or associated organic compounds. Conversely, we expected ^15^N supplied as urea to a root‐free substrate compartment (SC) to be recovered in plant biomass after the 3‐month field incubation period, indicating fungal‐mediated transfer from the SC back to plants. We further hypothesized that (2) microbial community composition and fungal–bacterial association patterns would differ among soil microhabitats along the gradient from rooted to root‐free hyphal compartments. Specifically, we expected microbial diversity to decrease toward the inner hyphal compartments due to increasing spatial separation from roots, dispersal constraints, and microhabitat filtering, resulting in fewer associations and a simplification of network structures. We anticipated a reorganization of fungal–bacterial associations toward a higher proportion of positive co‐occurrences, reflecting a stronger structuring role of fungi in shaping community assembly in hyphae‐dominated habitats.

## Materials and Methods

### Design of the hyphoboxes

Ingrowth cores (‘hyphoboxes’) were constructed from polyvinyl chloride (PVC) tubes that were open to both sides and contained five compartments filled with sterilized soil (Fig. 1, Supporting Information Fig. S1). Two outer (mycor)rhizosphere compartments (RCs) were separated from the adjacent soil, in the following referred to as bulk soil (BS), by 500‐μm nylon meshes (SEFAR‐NITEX, Thal, Switzerland), making them accessible to both plant roots and microbes. Further toward the hyphobox center, two hyphosphere compartments (HCs) were separated by 50‐μm nylon meshes preventing root ingrowth but allowing microbial colonization. A central SC was separated by 50 μm meshes and amended with root litter and ^15^N‐labeled urea. Air gaps of 4 mm were created between the SC and HCs with additional plastic rings to restrict transport of labeled material with the soil solution and ensure that only fungal hyphae and their associated bacteria could cross this air gap. Each compartment had an inner diameter of 7 cm and a height of 1 cm, corresponding to a volume of 38.5 cm^3^. Notably, in contrast to the soil filled into all hyphobox compartments (RC, HC, and SC), which had been sterilized before burial, the BS consisted of unsterilized grassland soil influenced by plant roots to varying degrees (i.e. partially rhizosphere‐affected soil).

**Fig. 1:**
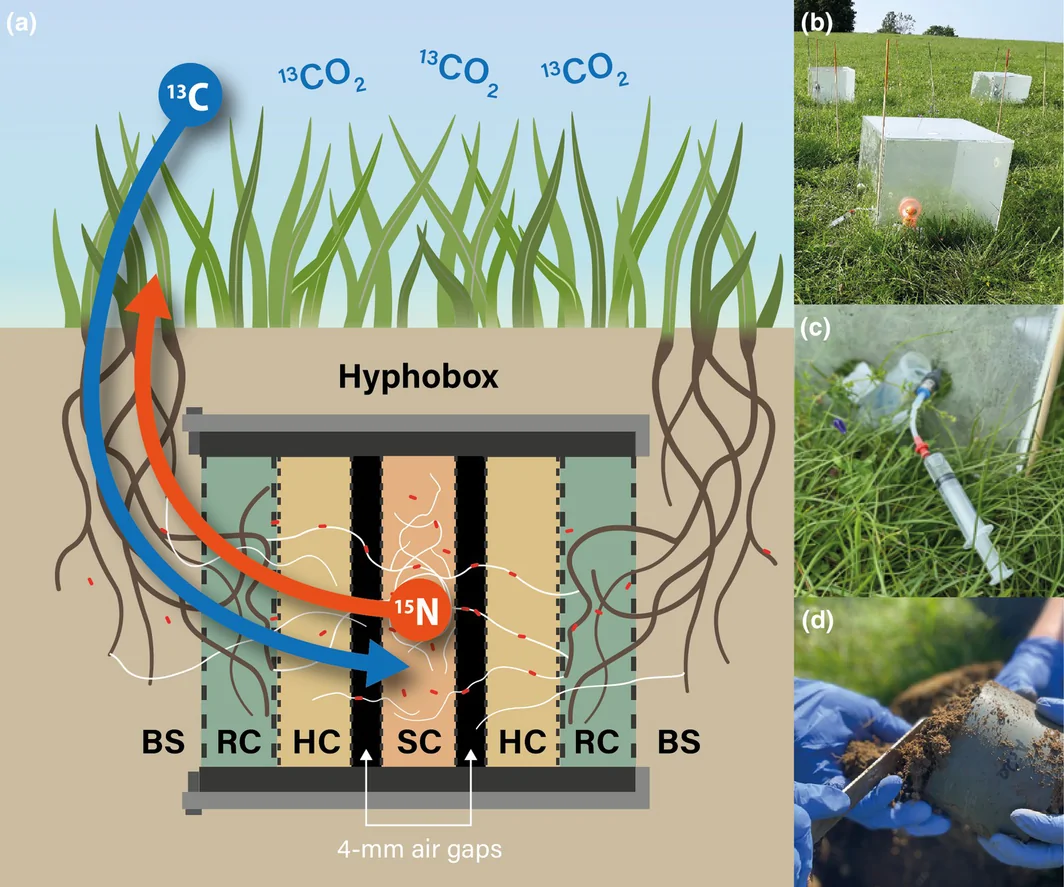
Overview of the experimental design and ^13^CO_2_ pulse‐labeling approach in the field. (a) Schematic representation of the hyphoboxes exposed to 17 cm soil depth. The hyphobox consists of a polyvinyl chloride (PVC) tube (outer and inner diameter: 10.4 and 9.1 cm, respectively; height: 7.5 cm) filled with different compartments (inner diameter: 7 cm; height: 1 cm; volume: 38.5 cm^3^): two outer (mycor)rhizosphere compartments (RCs), enclosed by 500‐μm meshes, which allow both root and microbial ingrowth; two hyphosphere compartments (HCs), enclosed by 50‐μm meshes, which exclude roots but allow hyphal access; and one central substrate compartment (SC), enclosed by 50‐μm meshes and separated from the HCs by 4‐mm air gaps. The SC is amended with ^15^N‐labeled urea to track its transfer via root‐free compartments to the plants. In parallel, the reciprocal transport of plant‐fixed ^13^C into the soil and its incorporation into microbial phospholipid and neutral fatty acids is examined. (b) Labeling chamber deployed at the grassland site. (c) Stepwise acid (H_3_PO_4_) addition was performed manually using a syringe connected by a hose to a falcon tube inside of the chamber, reacting with ^13^C‐labeled NaHCO_3_ to release CO_2_. (d) Sampling of a hyphobox together with adjacent bulk soil (BS).

### Hyphobox preparation and ^15^N labeling of the substrate compartment

The study was conducted in a temperate grassland of the Schwäbische Alb Exploratory, part of the German Biodiversity Exploratories (Fischer *et al*., 2010), in southwest Germany (48.40°N, 9.51°E). Soil for filling the hyphoboxes was collected on 11 March 2021, to a depth of 10 cm adjacent to the experimental plot where the hyphoboxes were subsequently installed. This site is a permanent grassland that is mown annually (one or two cuts per year), was historically subject to rotational sheep grazing (until 2016), and in recent years (2014, 2017, 2020, and 2021) received organic fertilization (Vogt *et al*., 2020). The grassland is dominated by *Bromus erectus* Huds., *Festuca rubra* L., *Trifolium repens* L., and *Ranunculus bulbosus* L., and belongs to the phytosociological class of *Festuco‐Brometea* (Oberdorfer, 2021). The soil at this site is classified as a Rendzic Leptosol (FAO classification; Ostrowski *et al*., 2021) with clay texture (71% clay, 27% silt, 2% sand; Schöning *et al*., 2012), a pH of 5.8 (Schöning, 2021), a total C content of 5.75%, and a total N content of 0.55%. The soil was sieved (< 5 mm), and recovered roots were washed. Soil and roots were dried at 80°C for 72 h as an initial sterilization step (Shih & Souza, 1978), and roots were chopped into fragments < 1 cm. To fill each SC, 39.73 g of dry soil was mixed with 0.27 g of dry root litter, corresponding to a typical root density of semi‐natural grasslands in the region (6.9 mg cm^−3^; Kandeler *et al*., 2019). For the urea‐N amendment, we applied 62.5 μg N g^−1^ soil, equivalent to *c*. 50 kg N ha^−1^ based on a bulk density of 0.8 g cm^−3^ and a soil depth of 10 cm. To each SC, we added 2.42 mg N containing 150 μg ^15^N in the form of 6 atom‐% ^15^N‐labeled urea dissolved in 16 ml autoclaved deionized water for the ^15^N‐labeled treatment, or the same amount of nonlabeled urea for the nonlabeled treatments. All other compartments were filled with 40 g of dry soil and adjusted to 60% water‐holding capacity with the same amount of 16 ml water. Assembled hyphoboxes were then wrapped in plastic film, sealed in zip‐lock bags, and stored at −20°C. To sterilize the entire hyphoboxes, they were irradiated with a total gamma dose of 75 kGy (25 kGy d^−1^ for three consecutive days) at BBF Sterilisationsservice GmbH (Kernen, Germany) and frozen at −20°C until burial (Trevors, 1996; McNamara *et al*., 2003).

### Hyphobox installation and ^13^CO_2_ pulse labeling

Thirty‐six hyphoboxes were buried on 15 April 2021, at 17 cm soil depth in a fully randomized layout, spaced 1 m apart within an 8 × 5 m subplot of the grassland experimental plot (Fig. S2). The experiment followed a factorial design of three labeling treatments × three sampling time points × four replicates. Labeling treatments included (1) dual ^13^C and ^15^N labeling (*n* = 12), (2) ^13^C‐only labeling (*n* = 12), and (3) unlabeled controls (*n* = 12). Each hyphobox was oriented with one opening facing northeast. For each treatment, four replicate hyphoboxes were harvested destructively 1, 3, and 7 d after ^13^CO_2_ pulse labeling, respectively. During installation, one ^13^C‐^15^N‐labeled hyphobox was damaged and replaced by a spare hyphobox without ^15^N labeling, reducing the replication of ^15^N data to *n* = 3 at the 3‐d time point. The specific analyses performed for each hyphobox are listed in Table S1.

To trace plant C allocation to hyphobox compartments and its incorporation into microbial phospholipid fatty acids (PLFAs) and neutral lipid fatty acids (NLFAs), we conducted *in situ* ^13^CO_2_ pulse labeling between 19 and 21 July 2021, 3 months after hyphobox burial, using transparent plexiglass chambers (Fig. 1b; 45 cm (L) × 45 cm (W) × 40 cm (H) = 81 000 cm^3^). Plant CO_2_ uptake was estimated beforehand at the same grassland site based on continuous CO_2_ measurements using a portable CO_2_ sensor, where plant biomass reduced chamber CO_2_ by *c*. 18 ppm min^−1^ (Fig. S3a), equivalent to 33.6 mg CO_2_ m^−3^ min^−1^. On each consecutive labeling day, eight chambers received ^13^CO_2_ during the morning hours (9:00 h–12:00 h CET). In parallel, CO_2_ concentration and temperature were manually monitored at 1–2‐min intervals for a control labeling chamber that was placed outside of the plot receiving unlabeled ^12^CO_2_ (Fig. S3b). The CO_2_ was generated inside of the chambers from 1.26 g NaHCO_3_ (either with 99 atom‐% ^13^C or with natural abundance) corresponding to 653.6 mg ^13^C–CO_2_ released. The NaHCO_3_ was stored within a 50‐ml falcon tube and CO_2_ was released by stepwise addition of 28% H_3_PO_4_ (v/v) via a hose connected to a syringe that was operated from outside of the chamber (Fig. 1c). When CO_2_ in the monitored control chamber dropped to *c*. 400 ppm, further acid additions were applied to all chambers until the NaHCO_3_ was consumed or 180 min had elapsed. Acid was added manually in 200 μl increments to maintain 380–800 ppm CO_2_. Fans inside of the labeling chambers ensured gas mixing.

### Hyphobox and plant biomass sampling

Hyphoboxes and plant shoot biomass were sampled 1, 3, and 7 d after pulse labeling (Table S1) to follow short‐term C flux dynamics. The plant biomass above each hyphobox was sampled from a 40 cm × 40 cm area previously covered by the labeling chamber and stored in paper bags. Plant biomass was later dried at 60°C for 72 h for C and N analyses. Hyphoboxes with surrounding soil attached were excavated with a spade. With a knife, we cut the BS to leave 1 cm of soil at each hyphobox opening (Fig. 1d). Hyphoboxes with attached soil were then sealed in zip‐lock bags and transported in cooling boxes to a lab fridge (4°C). From four selected hyphoboxes collected on Days 1 and 7 (one ^13^C‐labeled and one unlabeled control d^−1^), small soil cores were taken from all intact compartments and the adjacent soil for NanoSIMS analysis using sliced 15‐ml polypropylene centrifuge tubes (LABSOLUTE®, Art. No. 7696712, Th. Geyer, Renningen, Germany; Fig. S1e). All remaining soil material was then sieved (< 2 mm) and the two RC, HC, and BS samples per hyphobox were combined into composite samples. Aliquots of soil samples for sequencing were filled into cryotubes and flash‐frozen in liquid N. Remaining soil material was frozen at −20°C in plastic bags. Tools were cleaned with ethanol between samples to avoid cross‐contamination.

### Determination of carbon and nitrogen contents and isotopic signatures of plant biomass, roots, and soil

Dry plant biomass was coarsely ground in a knife mill and homogenized for each hyphobox. A subsample of the coarsely ground plant biomass, as well as aliquots of dry root samples from each RC, soil from the BS, and soil from each individual compartment of every hyphobox were then ground in a ball mill (Retsch, Haan, Germany). Approximately 2 mg of plant and root biomass and 20 mg of soil sample were weighed into 5 × 9 mm tin capsules (IVA Analysentechnik GmbH) and measured for total C, total N, as well as for ^13^C and ^15^N isotopic signature by dry combustion using an elemental analyzer (EA, Euro EA 3000; Euro Vector, Milan, Italy) coupled with an isotope ratio mass spectrometer (IRMS, Delta Plus XP; Thermo Finnigan MAT, Bremen, Germany) as described by Müller *et al*. (2016). Subsequently, isotope excess in soil and plant biomass relative to unlabeled control treatments was calculated as follows:

(Eqn 1) ExcessC13orN15=AT%sample−AT%control100×contentCN100×weightsample

where AT%_sample_ is the atom‐% ^13^C or ^15^N in labeled samples (soil, shoot biomass, or root biomass), and AT%_control_ is the atom‐% ^13^C or ^15^N in the unlabeled control, content_CN_ is the C or N concentration (%) of the respective sample, and weight_sample_ is the total dry mass of the sample. For plant shoots and roots, excess ^13^C and ^15^N were calculated for the total recovered biomass. For soil material within compartments and bulk soil samples, excess isotope amounts were calculated according to the mirrored hyphobox design: 80 g (two compartments, 40 g each) for the BS, RC and HC, and 40 g (one compartment) for the SC.

### Determination of phospholipid and neutral fatty acids and their ^13^C incorporation

PLFAs and NLFAs were extracted from the soil samples of the unlabeled and ^13^C‐only‐labeled treatments, following the methods of White *et al*. (1979) and Frostegård *et al*. (1991). Briefly, lipids were extracted from 4 g of fresh soil using 18.4 ml of Bligh & Dyer solution (chloroform, methanol and citrate buffer at pH 4; 1 : 2 : 0.8, v/v/v) on a horizontal shaker at 250 rpm for 2 h. Subsequent steps followed Ruess *et al*. (2007). Lipids were fractionated into neutral lipids, glycolipids, and phospholipids using silicic acid solid‐phase extraction columns (Bond Elut SI, 500 mg, 3 ml; Agilent Technologies, Santa Clara, CA, USA) with chloroform, acetone, and methanol as eluents, respectively (Zelles, 1999). Glycolipid fractions were discarded. Methanolysis of PLFAs and NLFAs was performed as described by Ruess *et al*. (2007). The resulting fatty acid methyl esters (FAMEs) were extracted, dissolved in isooctane, and analyzed using an Agilent 8860 gas chromatograph coupled with a 5977B mass selective detector (MSD; Agilent, Santa Clara, CA, USA). Peak identification and quantification were performed using a bacterial FAME mix (Sigma‐Aldrich) and individual FAME standards for calibration.

The PLFAs i15:0, a15:0, i16:0, and i17:0 were used as biomarkers for Gram‐positive bacteria, while cy17:0 and cy19:0 served as indicators of Gram‐negative bacteria. The PLFA 16:1ω7 was included as a general bacterial marker (Frostegård & Bååth, 1996; Zelles, 1999; Ruess & Chamberlain, 2010). Total bacterial PLFAs were calculated as the sum of Gram‐positive, Gram‐negative, and 16:1ω7 PLFAs. The PLFA 18:2ω6,9 was used to represent fungi (mainly saprotrophs in the studied grassland). Total microbial PLFAs were determined by summing all bacterial and fungal biomarker PLFAs. The NLFA 16:1ω5 was used as a biomarker for AMF (Ruess & Chamberlain, 2010).

For δ^13^C determination, PLFA and NLFA samples were fractionated using Ag^+^‐SPE cartridges (6 ml; Supelco, Palo Alto, CA, USA) following the method of Kramer & Gleixner (2008). This fractionation step was necessary to prevent overlapping peaks during analysis on a GC‐C‐IRMS system. A stepwise elution with 6 ml of n‐hexane containing increasing concentrations of acetone (99 : 1, 96 : 4, 90 : 10, and 0 : 100; v/v) was used to separate FAMEs into four fractions. The first fraction contained saturated FAMEs, the second and third fractions monoenoic *trans* and *cis* FAMEs, respectively, and the fourth fraction dienoic FAMEs. For isotopic analysis, the first and fourth fractions of PLFAs, and the third fraction of NLFAs, were retained, evaporated under a N stream, and re‐dissolved in 70 μl isooctane (Müller *et al*., 2016). δ^13^C values of PLFAs and NLFAs were measured using an HP 6890 gas chromatograph (Agilent) coupled via a Combustion III interface (Thermo Finnigan, Carlsbad, CA, USA) to a Delta Plus XP IRMS (Thermo Finnigan MAT, Hamburg, Germany), following the procedure described by Müller *et al*. (2016). Fatty acid peaks were identified by comparison with known standards.

During the methylation step, an additional C atom of known δ^13^C value (−40.23‰) is added to the fatty acid, which was accounted for according to Eqn 2.

(Eqn 2) $$\delta^{13}C_{\text{PLFA}}=\frac{\left(C_{\text{PLFA}}+1\right)\times \delta^{13}C_{\text{FAME}}-\delta^{13}C_{\text{MeOH}}}{C_{\text{PLFA}}}$$

where δ^13^C_FAME_ is the measured δ^13^C value of the FAME, C_PLFA_ and δ^13^C_PLFA_ refer to the number of C atoms and the δ^13^C value of the PLFA (or NLFA), respectively, and δ^13^C_MeOH_ refers to the δ^13^C value of the methanol used for methylation (Butler *et al*., 2003). The δ^13^C_PLFA_ values were subsequently converted to atom‐% based on the ^13^C/^12^C ratio of the VPDB standard (0.0111802).

### DNA extraction and amplicon sequencing

DNA was extracted from 0.3 g of soil frozen at −20°C following the manufacturer's protocol of the FastDNA™ SPIN Kit for Soil (MP Biomedicals, Santa Ana, CA, USA). DNA concentrations were quantified fluorometrically using the Quant‐iT PicoGreen dsDNA Assaykit (Thermo Fisher Scientific Inc., Waltham, MA, USA/Life Technologies GmbH, Darmstadt, Germany), following the manufacturer's guidelines. The amplification of the bacterial 16S rRNA gene was conducted targeting the V4 region with the primer pair 515F (Parada *et al*., 2016) and 806R (Apprill *et al*., 2015). For fungal assessment, the ITS 3 and ITS 4 primers were used (Tedersoo *et al*., 2015). One reaction mix (25 μl) contained 1 μl of extracted DNA (5 ng μl^−1^, or DEPC‐treated water for the negative control), 12.5 μl NEB Next High‐Fidelity Master Mix (New England Biolabs, Ipswich, MA, USA), 0.5 μl of each primer (at 10 pmol μl^−1^), 2.5 μl of 3% BSA, and 8 μl DEPC‐treated water. The reaction was initialized at 98°C for 60 s followed by denaturation (98°C, 10 s) within 23 cycles for 16S and 27 cycles for ITS, annealing (55°C, 30 s), and elongation (72°C, 30 s). Subsequently, a final elongation phase of 5 min (72°C) was performed. PCR products were purified using MagSi‐NGSPrep Plus Magnetic Beads (Steinbrenner Laborsysteme, Wiesenbach, Germany) with a ratio of PCR product and bead solution of 1 : 0.8. Quality assessment was performed via a Fragment Analyzer (Agilent, Waldbronn, Germany) using the DNF‐473 Standard Sensitivity NGS Fragment Analysis Kit (1–6000 bp). For indexing via Nextera® XT Index Kit v2 (Illumina, San Diego, CA, USA), all PCR products were diluted to 10 ng and 1 μl was used. Indexed PCR products were purified and quality‐checked as described above. Sample concentration was adjusted to 4 nM with DEPC water, and all samples were pooled equimolarly. The samples were sequenced on an Illumina MiSeq device, using the MiSeq Reagent Kit v3 (600 Cycle), generating paired‐end reads of 300 bp (Illumina).

### Bioinformatic processing

Adapter sequences were removed using fastp v.0.23.4 (Chen *et al*., 2018). Bacterial (16S) and fungal ITS sequence reads were processed separately using the DADA2 pipeline (dada2 package v.1.30.0; Callahan *et al*., 2016) in R v.4.3.2 (R Core Team, 2023) on a high‐performance computing cluster at the Helmholtz Institute in Munich. For bacterial sequences, the standard dada2 workflow (v.1.16; https://benjjneb.github.io/dada2/tutorial.html) was applied. Forward and reverse reads were trimmed and filtered based on per‐base quality profiles to 250 bp and 210 bp, respectively, with 19 bp (forward) and 20 bp (reverse) removed from the 5′ ends. The maximum expected error rate (maxEE) was set to 3 for forward reads and 4 for reverse reads. Fungal ITS sequences were processed using the ITS‐specific dada2 workflow (v.1.8; https://benjjneb.github.io/dada2/ITS_workflow.html). Residual primer sequences were removed using cutadapt v.4.9 (Martin, 2011). Forward and reverse reads were trimmed and filtered to 250 bp and 210 bp, respectively, with a maxEE threshold of 4 for both read directions.

Following quality filtering, reads from both datasets were denoised and amplicon sequence variants (ASVs) were inferred using dada2. Read retention at each step of the dada2 pipeline is provided in Table S2 (16S rRNA gene) and S3 (ITS). Taxonomic assignment of 16S ASVs was performed using the assignTaxonomy and addSpecies functions against the SILVA database v.138.2 (Quast *et al*., 2012). Fungal ASVs were taxonomically classified using the UNITE database v.10.0 (Abarenkov *et al*., 2025). Separate phyloseq objects for 16S and ITS datasets were constructed using the phyloseq R package v.1.46.0 (McMurdie & Holmes, 2013). Contaminants, negative controls, and chloroplast and mitochondrial sequences were identified and removed using the decontam R package v.1.22.0 (Davis *et al*., 2018). ASVs represented by a single read were excluded from downstream analyses.

### NanoSIMS imaging

Soil cores intended for NanoSIMS analysis were placed in acetone‐resistant plastic containers with mesh bottoms to allow drainage of fixation and dehydration solutions. Samples were fixed by slowly adding Karnovsky fixative (Karnovsky, 1965) until fully submerged. Subsequently, the samples were placed in a desiccator under vacuum (0.8 bar) for 24–48 h to facilitate infiltration of the fixative (Nunan *et al*., 2001). The fixative was then gradually replaced with increasing concentrations of acetone (10, 25, 50, 75, and 100%), maintaining each step under vacuum for 48 h. The final 100% acetone step was repeated twice, with the last step lasting 2 wk. For embedding, an Araldite 502 epoxy resin was used (Finck, 1960; Herrmann *et al*., 2007). The resin was first mixed 1 : 1 with acetone and applied under vacuum for 24 h. Subsequently, pure Araldite was added. The embedded resin block was cut into 3‐mm thick discs in a radial direction using a low‐speed saw (Allied Techcut 4; Allied High Tech Products, Cerritos, CA, USA). The sections were glued onto a glass stub, then ground and polished with diamond disks to a final thickness of < 1 mm and a flat surface suitable for NanoSIMS analysis. Regions of interest at root‐soil interfaces and other spots possibly hosting soil–microbe interactions were identified for subsequent NanoSIMS measurements using a Scanning Electron Microscope (JEOL JSM‐5900LV; JEOL Ltd, Tokyo, Japan). NanoSIMS analysis was performed using a NanoSIMS 50 l instrument (Cameca, Gennevilliers, France) at the Technical University of Munich. Measurements were conducted using a 16 keV Cs^+^ primary ion beam with simultaneous detection of seven ion channels, namely ^16^O^−^, ^12^C^12^C^−^, ^13^C^12^C^−^, ^12^C^14^N^−^, ^12^C^15^N^−^, ^31^P^−^, and ^27^Al^16^O^−^. For each image, the field of view spanned 30 × 30 or 35 × 35 μm, which were recorded as multilayer image stacks with a 256 × 256‐pixel image resolution, a dwell time of 1 ms per pixel and 45 repeated scans. Obtained image data were dead‐time corrected, auto‐tracked, and accumulated utilizing the OpenMIMS plugin for fiji (Gormanns *et al*., 2012; Schindelin *et al*., 2015; Hu *et al*., 2024). The corresponding individual secondary ion images for all measured ion species are provided in Fig. S4. To analyze the allocation of plant‐derived ^13^C and urea‐derived ^15^N, we conducted a multichannel machine‐learning segmentation (Inagaki *et al*., 2020). First, we differentiated resin‐filled pores (high in ^12^C_2_^−^), mineral‐dominated regions (high in ^16^O^−^), and OM‐dominated regions (high in ^12^C^14^N^−^). Subsequently, ^13^C‐ and ^15^N‐enriched spots were identified by calculating the ^13^C and ^15^N enrichment according to Shabtai *et al*. (2024), accounting for the fact that C was detected as C_2_ cluster ions. The resulting enrichment values were scaled to a range of 0–3% to standardize the images for the supervised machine‐learning segmentation. Enrichment values ≥ 3% were treated as equivalent (i.e. capped at this upper threshold) during scaling. The segmentation masks were then combined, and mean ^13^C or ^15^N enrichment within a given structural region was calculated as an area‐weighted average across all pixels assigned to that region. To assess the composition and reliability of classified segments, mean secondary ion counts per segment type (nonenriched and enriched resin, mineral, and OM) were extracted (Fig. S5). In total, 31 measurements were distributed across all compartments, that is nine in BS, six in RC, eight in HC, and four in SC samples, respectively, plus four unlabeled controls. Given the cost and time constraints of NanoSIMS, the number of measurements was limited, and we do not claim quantitative representativeness. The four images shown were selected because they displayed detectable ^13^C and/or ^15^N enrichment, whereas most other spots showed no enrichment above natural abundance. The primary aim of these images was therefore to visualize spatial patterns of isotope‐label distribution across the heterogeneous organo‐mineral soil matrix at the microscale, rather than to provide quantitative, compartment‐wide estimates of isotope enrichment.

### Data and statistical analysis

Data were analyzed separately for plants, roots, and soil. Linear mixed‐effects models were constructed with the lme4 package v.1.1‐33 (Bates *et al*., 2015) in R v.4.3.0 and used for soil‐related parameters (soil water content, excess ^13^C and ^15^N, PLFA and NLFA contents and their δ^13^C values, Simpson and Shannon diversity), including hyphobox as a random effect to account for the nested structure of compartments within each hyphobox. For shoot and root biomass, linear models were applied since no random structure was required. Categorical fixed effects included compartment (BS, RC, HC, and SC), time (1, 3, and 7 d after ^13^CO_2_ pulse labeling), and treatment (^13^C‐^15^N‐labeled, ^13^C‐only‐labeled, and unlabeled) as well as their interactions in the initial models. Model simplification was based on ANOVA using the R function Anova (car package v.3.1‐2; Fox & Weisberg, 2019), with nonsignificant interactions sequentially removed to obtain the most parsimonious model structure. To specifically assess δ^13^C signatures of microbial lipid markers, differences between labeled and unlabeled treatments were tested within each time × compartment combination. Model assumptions were checked by visual inspection of residual plots for normality and homogeneity of variance. Post hoc pairwise comparisons were conducted using the emmeans package v.1.8.6 (Lenth & Piaskowski, 2025). Detailed model outputs are provided in Tables S4 and S5.

Differences in bacterial and fungal community composition among BS and hyphobox compartments were tested by performing PERMANOVA with the adonis2 function (R package vegan v.2.6‐4; Oksanen *et al*., 2022) based on Bray–Curtis dissimilarities of relative abundance data, including the hyphobox number as a strata term to account for the nested experimental design. Nonmetric multidimensional scaling (NMDS) was then performed to visualize differences in community compositions among samples using the ordinate function (phyloseq package). Microbial diversity was assessed as Simpson and Shannon diversity using the R function estimate_richness (phyloseq package). Differential abundance of fungal and bacterial phyla between compartments was tested using deseq2 v.1.42.1 (Love *et al*., 2014). Log_2_‐fold changes (LFCs) were shrunk with lfcShrink. Phyla were considered significantly enriched or depleted when *P* < 0.05 and |LFC| > 1.5, reflecting biologically meaningful effect sizes.

Cross‐domain network analysis was performed according to the cross‐domain NetCoMi framework with association matrix creation with spieceasi v.1.1.3 (Tipton *et al*., 2018; Kurtz *et al*., 2025), followed by network construction and analysis using netcomi v.1.2.0 (Peschel *et al*., 2021) in R v.4.4.1. ASVs were filtered to retain those present in at least five out of eight samples per compartment. A pseudocount of 1 was added to the filtered count data, followed by compositional transformation. For each compartment, an association matrix was inferred from the combined 16S rRNA gene and ITS count matrices using the multi.spiec.easi function. Model selection was performed with nlambda = 70, lambda.min.ratio = 10^−3^, and pulsar stability selection (thresh = 0.05, rep.num = 50). One network per compartment was constructed from the resulting association matrices. Network hubs were identified based on eigenvector centrality using a 0.90 quantile threshold (Ruhnau, 2000), and community structure was inferred using the fast greedy clustering algorithm (Clauset *et al*., 2004). Networks were visualized using the circlize package v.0.4.17 (Gu *et al*., 2014). For visualization, only positive and negative edges representing fungal–bacterial associations were retained and aggregated at the phylum level. All other plots were produced using the ggplot2 package v.4.0.0 (Wickham, 2016).

## Results

### ^13^C and ^15^N enrichment in plants and soil

Plant shoot biomass in the 40 cm × 40 cm area above each hyphobox covered by the labeling chamber and root biomass in the RCs did not differ among the three labeling treatments. However, both shoot biomass (*F*_2,33_ = 13.75, *P* < 0.001) and root biomass (*F*_2,33_ = 14.95, *P* < 0.001) varied significantly among sampling days. Hyphoboxes sampled 7 d after the time of the ^13^CO_2_ pulse labeling had significantly higher shoot biomass (48.2 ± 18.3 g; mean ± SD) than those sampled after 1 (25.0 ± 4.8 g) and 3 d (33.2 ± 2.1 g), whereas root biomass was lower at Day 7 (1.4 ± 0.8 g) than at Days 1 (5.5 ± 3.3 g) and 3 (7.3 ± 3.1 g; Fig. S6). Because sampling was destructive, these differences among sampling days represent variation among independently sampled field positions rather than temporal changes within the same plant community. Despite the temporal differences in plant shoot and root biomass, their ^13^C excess (Fig. 2a,b) and ^15^N excess (Fig. 2d,e) remained stable across sampling days. On average, plant shoots contained 12.7 ± 4.0 mg ^13^C and 20.8 ± 4.3 μg ^15^N excess, and roots 173.3 ± 60.0 μg ^13^C and 4.5 ± 0.9 μg ^15^N excess. The corresponding isotopic signatures of all sample types are given as atom‐% in Fig. S7.

**Fig. 2:**
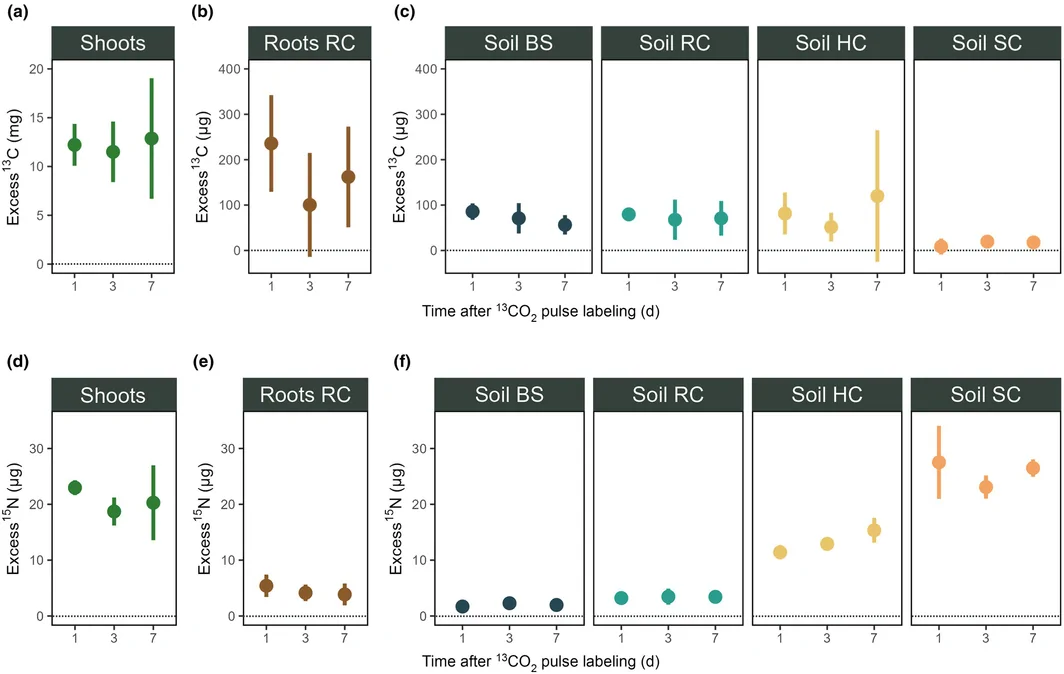
Excess ^13^C and ^15^N in plants and soil in labeled treatments relative to the unlabeled control. Excess ^13^C in (a) recovered plant shoot biomass, (b) recovered plant root biomass of the (mycor)rhizosphere compartment (RC), and (c) the bulk soil (BS) and soils of the different hyphobox compartments, that is the RC, hyphosphere compartment (HC), and substrate compartment (SC). Excess ^15^N in (d) the shoot biomass, (e) root biomass of the RC, and (f) the BS, RC, HC, and SC soils. Soil excess values were calculated based on the mirrored hyphobox design and are expressed per total soil mass of 80 g for the BS, RC, and HC (two compartments of 40 g each) and 40 g for the SC. Dots show means and bars SD, where each mean represents *n* = 8 observations for ^13^C excess and *n* = 4 for ^15^N excess, except for ^15^N at Day 3 where *n* = 3. The dotted line indicates zero excess (i.e. natural abundance level). All ^13^C and ^15^N excess values were significantly different from zero (one‐sample *t*‐tests, all *P* < 0.001). Note the difference in units between plant shoot ^13^C excess (mg) and all other panels (μg).

Similar to the plant isotope parameters, soil ^13^C and ^15^N excess did not differ among sampling days but varied significantly among BS and hyphobox compartments (^13^C excess: χ^2^(3) = 30.1, *P* < 0.001; ^15^N excess: χ^2^(3) = 688.9, *P* < 0.001). Soil ^13^C excess was comparable in the BS, RC, and HC (overall mean 76.0 ± 57.3 μg ^13^C) but was about fivefold lower in the SC (with 15.3 ± 14.4 μg ^13^C; Fig. 2c). Conversely, soil ^15^N excess was highest in the SC (25.9 ± 4.2 μg ^15^N) and decreased significantly in the HC (13.3 ± 2.3 μg ^15^N) and RC (3.4 ± 0.8 μg ^15^N). The RC and BS (2.0 ± 0.6 μg ^15^N) showed similarly low levels. Soil water content also differed significantly among compartments (χ^2^(3) = 144.1, *P* < 0.001), being roughly 1.2‐fold higher in the BS than in the hyphobox compartments (Fig. S8).

Overall, of the 150 μg ^15^N initially added to the SC at the start of the experiment, 46.6% was recovered after 3 months of hyphobox exposure: 16.9% was contained in plant material (13.9% in plant shoots and 3.0% in roots of the RC), while a greater proportion of 29.7% remained in the soil (17.3% in the SC, 8.9% in the HC, 2.3% in the RC, and 1.3% in the BS).

### Phospholipid and neutral fatty acids and their ^13^C incorporation

The PLFA contents did not differ among labeling treatments or sampling days but varied strongly among compartments (saprotrophic fungi: χ^2^(3) = 463.8, *P* < 0.001; Gram‐negative bacteria: χ^2^(3) = 1754.1, *P* < 0.001; Gram‐positive bacteria: χ^2^(3) = 3704.3, *P* < 0.001; Fig. 3a). Fungal and bacterial PLFAs were approximately twofold more abundant in the BS than in the RC and generally further declined slightly toward the HC and SC (Fig. 3a). The AMF biomarker NLFA 16:1ω5 also varied across compartments (χ^2^(3) = 63.1, *P* < 0.001), being similarly low in the BS and SC but significantly higher in the RC and HC (Fig. 3a). NLFA contents additionally differed among sampling days (χ^2^(3) = 9.4, *P* = 0.009) and showed a significant compartment × time interaction (χ^2^(6) = 17.8, *P* = 0.007): contents were higher in hyphoboxes sampled 7 d compared with 1 d after ^13^CO_2_ pulse labeling in all three hyphobox compartments, and higher at Day 3 than Day 1 in the HC and SC (Fig. S9). In addition, a significant compartment × treatment interaction (χ^2^(3) = 11.2, *P* = 0.011) indicated greater AMF NLFA abundance under ^13^C‐labeling compared with unlabeled controls in the SC.

**Fig. 3:**
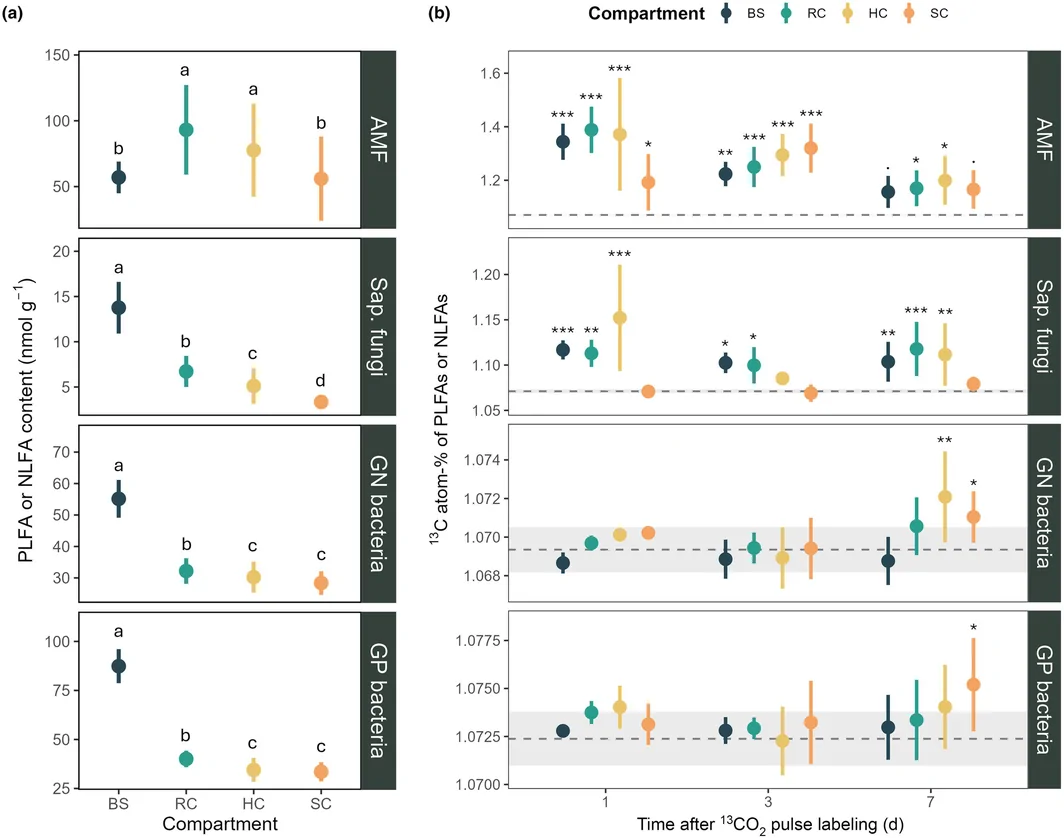
Microbial fatty acid contents and their ^13^C incorporation. (a) Average contents of phospholipid fatty acids (PLFAs) for saprotrophic (sap.) fungi, Gram‐negative (GN) as well as Gram‐positive (GP) bacteria, and of neutral lipid fatty acids (NLFAs) of arbuscular mycorrhizal fungi (AMF) in the bulk soil (BS) and hyphobox compartments, that is the (mycor)rhizosphere compartment (RC), the hyphosphere compartment (HC), and the substrate compartment (SC) across timepoints. Dots show means and bars SD, where each mean represents *n* = 24 observations, while letters denote significant differences (*P* < 0.05) according to linear mixed‐effects models. (b) ^13^C atom‐% of PLFAs or NLFAs of microbial groups in the BS and hyphobox compartments 1, 3, and 7 d post‐^13^CO_2_ pulse labeling in the ^13^C‐labeled treatment. Dots show means and bars SD, where each mean represents *n* = 4 observations, except for saprotrophic fungi in the HC and SC at Day 3, where *n* = 3. Symbols indicate statistical differences from ^13^C atom‐% values in the unlabeled control treatment within the same time × compartment combination, based on linear mixed‐effects models and Tukey‐adjusted pairwise comparisons (emmeans; Supporting Information Table S5). Significance levels are denoted as follows: ***, *P* < 0.001; **, *P* < 0.01; *, *P* < 0.05; ., *P* < 0.1; gray dotted lines and light gray areas in the background indicate mean values and SD in the unlabeled control treatment averaged across compartments and timepoints across *n* = 48 observations for both fungal groups and *n* = 47 observations for both bacterial groups.

In unlabeled controls, ^13^C atom‐% of PLFAs and NLFAs were similar across all microbial groups and averaged 1.07 ± 0.001 across compartments and sampling days. One day after ^13^CO_2_ pulse labeling, ^13^C incorporation was most pronounced in fungi (Fig. 3b): the AMF NLFA exhibited strong initial ^13^C enrichment across all compartments (Δ^13^C atom‐% between labeled and unlabeled treatment: BS, +0.275; RC, +0.319; HC, +0.301; SC, +0.121). Over Days 3 and 7, ^13^C enrichment in the NLFA declined to less than half of initial values in most compartments, whereas in the SC, ^13^C atom‐% values slightly increased until Day 3 before decreasing. The saprotrophic fungal PLFA also showed significant ^13^C enrichment 1 d after labeling in the BS (+0.047), RC (+0.041), and HC (+0.081), but not in the SC. Its ^13^C atom‐% values then remained relatively stable throughout the 7‐d period. Bacterial PLFAs showed no early ^13^C incorporation. However, by Day 7, slight but significant increases were detected in Gram‐negative bacteria in the HC (+0.003) and SC (+0.002), and in Gram‐positive bacteria in the SC (+0.003; Fig. 3b).

### NanoSIMS imaging

Based on NanoSIMS measurements at the microscale, the imaged sections revealed a heterogeneous soil matrix with a distinct spatial distribution of organic and mineral components. The mineral soil matrix was predominantly Al‐dominated (^27^Al^16^O^−^), indicating aluminosilicates, such as clay minerals, whereas P (^31^P^−^) was distributed in discrete, μm‐sized hotspots (Fig. S10). NanoSIMS imaging revealed ^13^C hotspots located centrally within a root of the BS, corresponding to the phloem/xylem region (Fig. 4a). A small (< 1 μm) hotspot of SC‐derived ^15^N enrichment was similarly localized within these central, vascular root structures in the BS (Fig. 4a), and further ^15^N enrichment was detected along a longitudinal root section in the RC (Fig. 4b). In the HC, ^15^N‐enriched hotspots occurred within the heterogeneous mineral soil matrix, without any associated root or hyphal structures. In the SC, hyphae‐like structures (*c*. 5 μm diameter) were identified, which were closely associated with ^15^N‐enriched hotspots in the adjacent soil matrix, whereas these were not found to contain plant‐derived ^13^C enrichment (Fig. 4d).

**Fig. 4:**
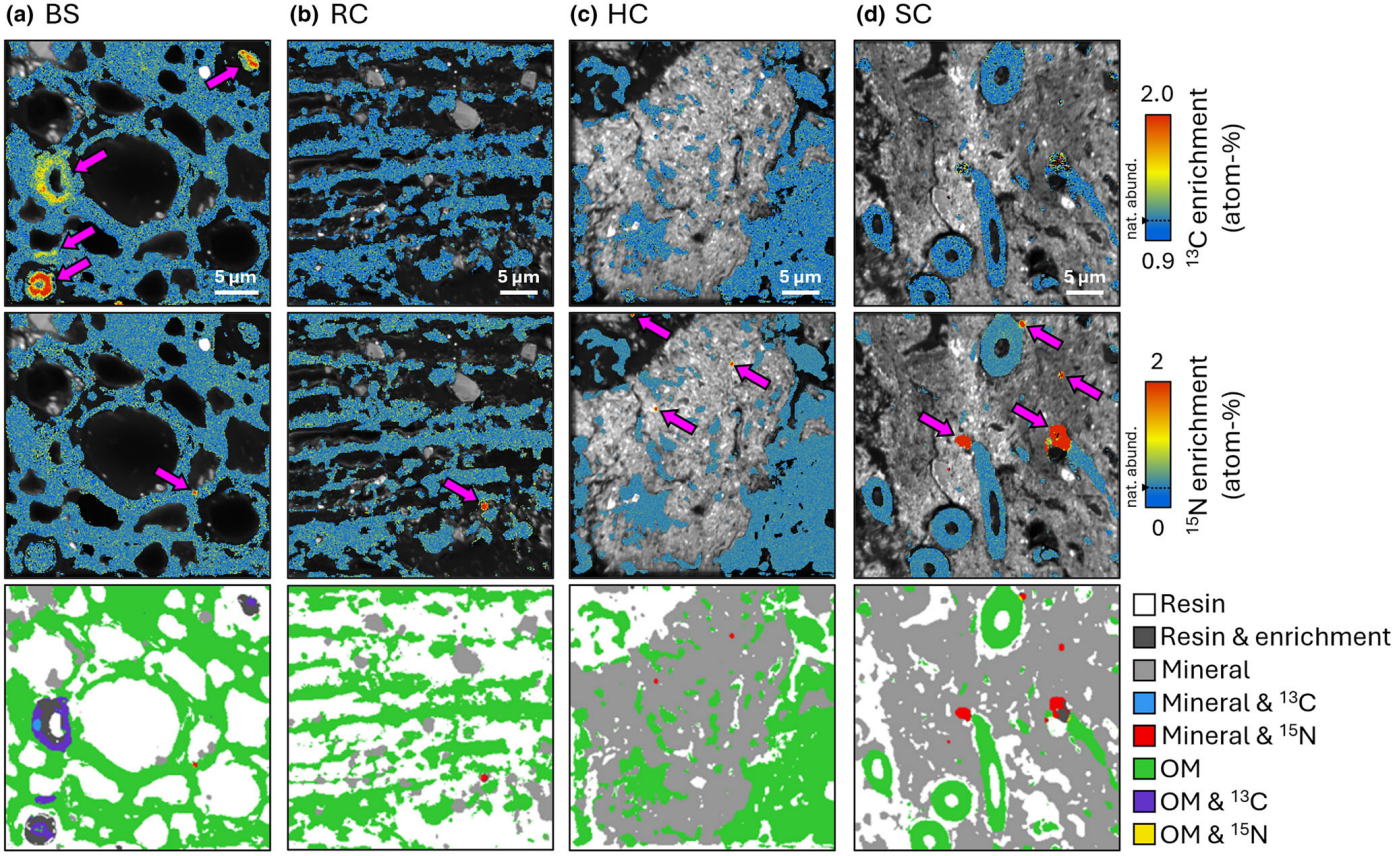
Nanoscale secondary ion mass spectrometry (NanoSIMS) images of ^13^C and ^15^N enrichment in plant roots and soil. Shown are (a) a transverse root section in the bulk soil (BS), (b) a longitudinal root section in the (mycor)rhizosphere compartment (RC), and (c) the soil matrix in the hyphosphere compartment (HC) 1 d after ^13^CO_2_ pulse labeling. (d) shows NanoSIMS images containing multiple cross‐sections of hyphae‐like structures in the substrate compartment (SC), 7 d after ^13^C labeling. For each column, the top and middle rows show gray‐scale SEM images overlaid with false‐color ^13^C and ^15^N enrichment (in atom‐%) of organic matter (OM), respectively; pink arrows highlight hotspots of isotopic enrichment. The bottom row shows a classification of structures into nonenriched embedding resin (white), enriched embedding resin (dark gray), nonenriched mineral‐dominated regions (light gray), ^13^C‐enriched mineral‐dominated regions (blue), ^15^N‐enriched mineral‐dominated regions (red), nonenriched OM‐dominated regions (green), ^13^C‐enriched OM‐dominated regions (purple), and ^15^N‐enriched OM‐dominated regions (yellow). The field of view spans 30 × 30 μm for (a) and 35 × 35 μm for (b–d), respectively.

### Fungal and bacterial diversity and community compositions across microhabitats

In total, 3953 968 paired‐end reads were obtained for the 16S rRNA gene and 2012 684 paired‐end reads for the ITS region across 38 samples (Tables S2, S3). After quality filtering and processing with the DADA2 pipeline, 2589 516 (16S) and 1336 139 (ITS) nonchimeric reads were retained, from which 5388 bacterial and 1933 fungal ASVs were inferred. Bacterial and fungal diversity metrics, that is Simpson diversity and Shannon diversity, declined progressively from the BS to the RC and HC (Fig. S11). Bacterial diversity showed a further reduction toward the SC, whereas fungal diversity remained comparable between the HC and SC. Fungal and bacterial community compositions differed significantly among the BS and the different hyphobox compartments (Fig. S12; PERMANOVA bacteria: *R* ^2^ = 0.70, *F*_3,28_ = 22.22, *P* = 0.001; PERMANOVA fungi: *R* ^2^ = 0.36, *F*_3,28_ = 5.17, *P* = 0.001). The greatest compositional shift in bacterial communities occurred when transitioning from the BS into the RC (Fig. 5a): several of the 15 overall most abundant bacterial phyla (*Bacteroidota*, *Bacillota*, *Fibrobacterota*, *Bdellovibrionota*, and *Patescibacteria*) were relatively enriched in the newly colonized RC as compared to the adjacent BS, whereas others (*Verrucomicrobiota*, *Acidobacteriota*, *Methylomirabilota*, and *Thermoproteota*) were relatively depleted (Figs 5a, S13). Between hyphobox compartments, further differences in relative abundances of bacterial phyla were minor: only *Thermoproteota* and *Methylomirabilota* continued to decrease from the RC toward the HC, with *Thermoproteota* increasing again toward the SC. Overall, the most dominant bacterial phyla across hyphobox compartments belonged to *Pseudomonadota* (on average 44% relative abundance across compartments), *Bacteroidota* (23%), *Actinomycetota* (10%), *Bacillota* (5%), *Planctomycetota* (4%), and *Verrucomicrobiota* (4%).

**Fig. 5:**
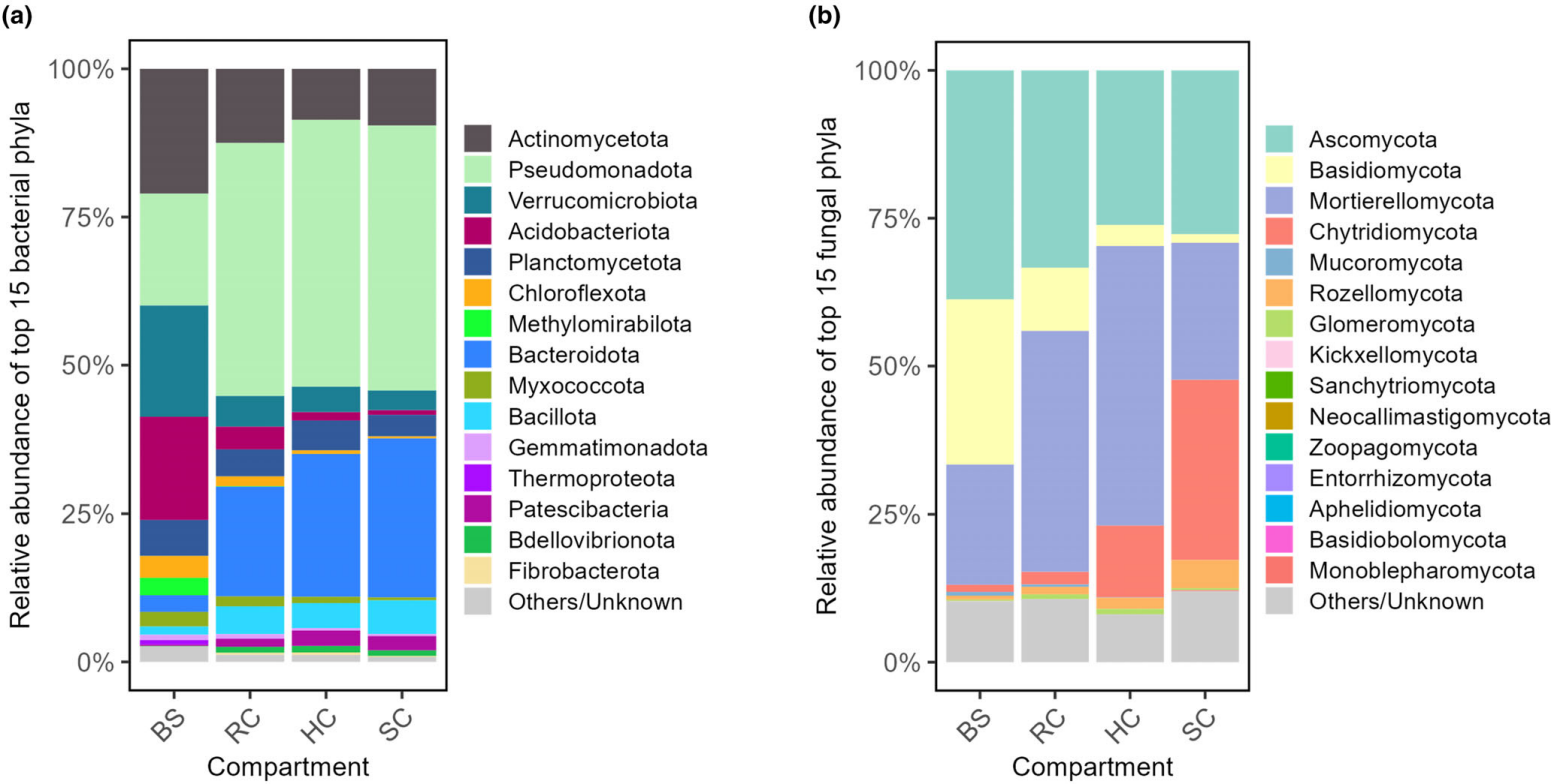
Relative abundance of the 15 most abundant bacterial and fungal phyla across soil compartments. Most abundant (a) bacterial and (b) fungal phyla in the bulk soil (BS) and hyphobox compartments, that is the (mycor)rhizosphere compartment (RC), the hyphosphere compartment (HC), and the substrate compartment (SC). Shown are mean values across *n* = 8 replicates for each compartment of the hyphoboxes sampled 1 d after the ^13^CO_2_ pulse labeling. The 15 most abundant bacterial phyla comprise 4923 of 5388 total ASVs and account for on average 98.6% of relative abundance across compartments. The 15 most abundant fungal phyla comprise 1401 of 1933 total ASVs and account for on average 89.7% of relative abundance across compartments.

The relative abundance of the 15 overall most abundant fungal phyla was overall less strongly affected when crossing from the BS into the RC (Fig. 5b). Only *Basidiomycota* showed a significant decrease from the BS toward the RC and continued to decrease in the HC (Fig. S14). Within the hyphoboxes, only a few additional shifts in fungal phyla occurred: *Chytridiomycota* strongly increased from the RC through the HC to the SC, while *Mucoromycota* consistently declined from the RC to the HC and the SC. Overall, the most abundant fungal phyla across hyphobox compartments belonged to *Mortierellomycota* (on average 37% across compartments), *Ascomycota* (29%), *Chytridiomycota* (15%), *Basidiomycota* (5%), and *Rozellomycota* (3%). AMF (phylum *Glomeromycota*) had a relatively stable relative abundance across BS, RC, and HC (on average 0.65%) and showed a slight decrease toward the SC (0.34%), which was generally in line with the pattern observed for the NLFA content.

### Cross‐kingdom correlation networks across microhabitats

Across compartments, 1250 bacterial and 165 fungal ASVs were included in the reconstructed networks, which were assigned to 32 bacterial and 8 fungal phyla (Fig. 6). Of these, 178 bacterial ASVs and 23 fungal ASVs were identified as network hubs, the vast majority of which were exclusive to a specific compartment. Only three bacterial ASVs occurred as hubs across a maximum of two compartments: one ASV classified as *Thermoflavimicrobium dichotomicum* was a hub in both the HC and SC networks, while two ASVs with limited taxonomic resolution, affiliated with the family *JG30‐KF‐CM45* and the order *Gaiellales*, were hubs in the RC and SC networks and the BS and RC networks, respectively. Along the gradient from BS through RC and HC to SC, the network size decreased markedly, with the number of hubs, nodes, and edges and the average degree continuously declining (Table 1). At the same time, the modularity, density, natural connectivity, and average path length increased, particularly to the SC, reflecting a reorganization from many bilateral toward fewer but more modular interactions (Table 1).

**Fig. 6:**
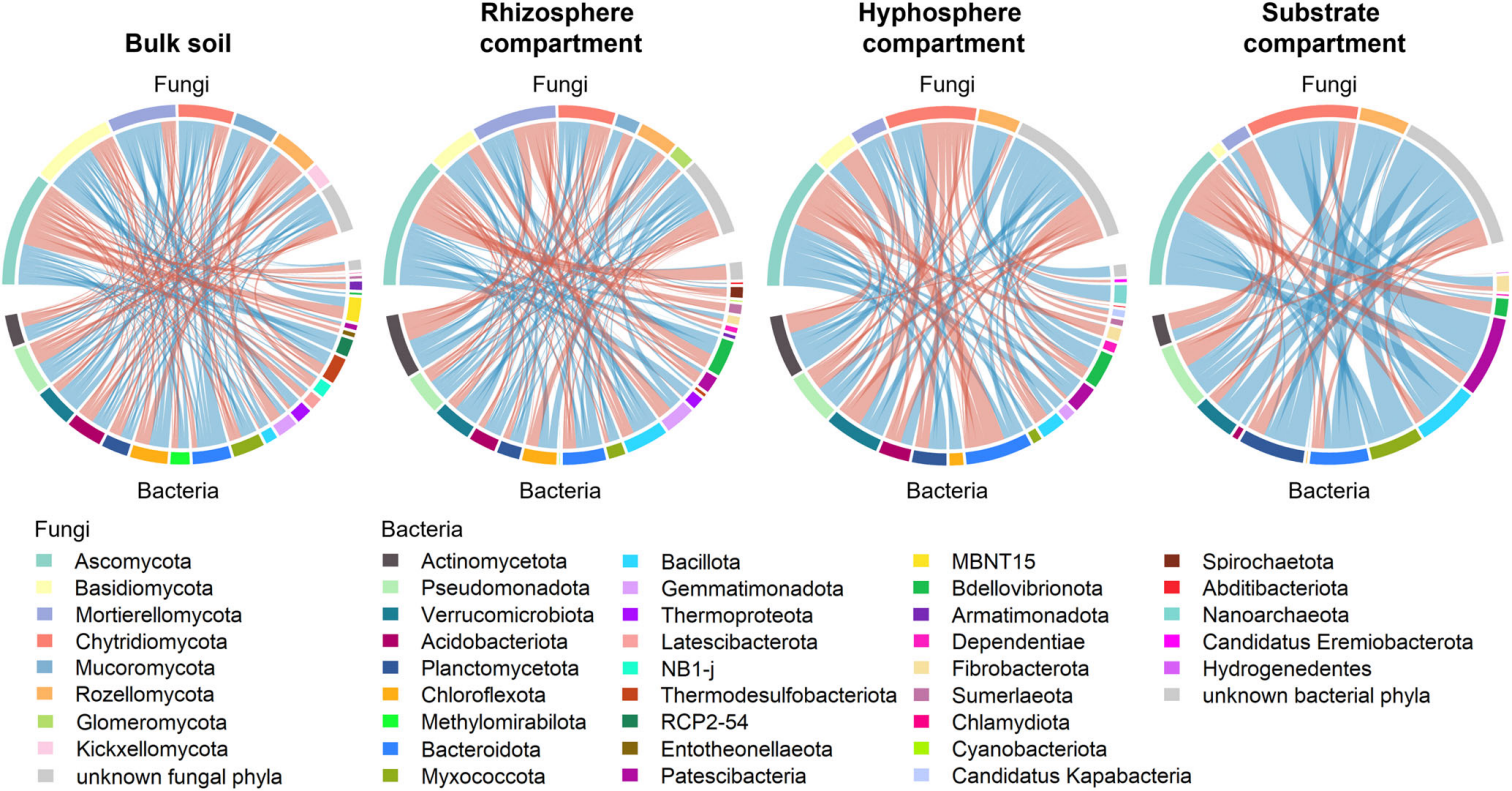
Chord diagrams showing the average strength of amplicon sequence variant (ASV)‐level associations between bacterial and fungal phyla within the adjacent bulk soil and hyphobox compartments, as inferred from network analysis (*n* = 8 per network). Connections represent mean association values aggregated across all cross‐kingdom ASV–ASV interactions linking each phylum pair. Blue chords indicate positive associations, while red chords indicate negative associations.

**Table 1:** Network topology metrics of cross‐domain fungal–bacterial association networks.

Network property	Bulk soil	Rhizosphere compartment	Hyphosphere compartment	Substrate compartment
Number of nodes	810	544	433	337
Bacterial nodes (%)	84	88	92	92
Fungal nodes (%)	16	12	8	8
Number of edges	5736	3068	2118	1225
Positive edges (%)	56.4	57.4	61.1	67.8
Number of fungal–bacterial edges	1460	629	269	172
Positive fungal–bacterial edges (%)	55.8	55.5	58.3	60.5
Degree (mean ± SD)	14.2 ± 2.6	11.3 ± 2.2	9.8 ± 1.7	7.3 ± 1.7
Density	0.017	0.021	0.023	0.022
Modularity	0.376	0.383	0.378	0.464
Natural connectivity	0.0022	0.0030	0.0035	0.0042
Average path length	2.13	2.11	2.10	2.30
Global clustering coefficient	0.166	0.139	0.121	0.123
Number of bacterial hubs	68	44	44	25
Number of fungal hubs	13	9	1	0

The proportion of positive edges in the overall networks, as well as within fungal–bacterial associations, increased from BS and RC toward the hyphal compartments HC and SC (Table 1). While *Ascomycota* maintained relatively stable proportions of positive vs negative associations with bacterial partners across compartments, other fungal groups exhibited more compartment‐specific association patterns: *Basidiomycota*, *Chytridiomycota*, and *Rozellomycota*, for instance, were characterized by a marked predominance of positive associations in the SC. For bacteria, positive associations across compartments mainly involved *Actinomycetota*, *Pseudomonadota*, *Verrucomicrobiota*, *Planctomycetota*, *Bacteroidota*, *Myxococcota*, *Bacillota*, and *Patescibacteria*, among others. For instance, the strongest positive associations in the SC were observed between *Lobulomycetales* (*Chytridiomycota*) and *LWQ8* (*Patescibacteria*), *Betamyces* (*Chytridiomycota*) and *Spirosomataceae* (*Bacteroidia*), as well as *Pseudopithomyces* (*Ascomycota*) and *Paenibacillaceae* (*Bacillota*).

With respect to AMF (*Glomeromycota*), only *Claroideoglomus* was detected in the RC network, which exhibited positive associations with representatives of *Acidobacteria* (*Vicinamibacteraceae*), *Bacillota* (*Thermoactinomyces daqus*), *Chloroflexota* (*Aggregatilineaceae*), *Planctomycetota* (*SH‐PL14*), *Pseudomonadota* (*Massilia*, *Pseudomonas*, *Pararhizobium*, *Brevundimonas*, *Tardiphaga robiniae*, and *Caulobacter*), and *Verrucomicrobiota* (*Pedosphaeraceae*). At the same time, we observed negative associations between *Claroideoglomus* and *Bacillota* (*Cohnella*), *Pseudomonadota* (*Steroidobacter*, *Arenimonas*, and *Reyranella*), *Verrucomicrobiota* (*Verrucomicrobiaceae*), and *Fibrobacterota* (*Fibrobacteraceae*).

## Discussion

Using compartmented ingrowth cores combined with isotope tracing and cross‐kingdom network analyses in a temperate grassland, we traced a reciprocal C–N exchange between plants and spatially distant soil microhabitats and resolved the microbial communities mediating it. Newly plant‐fixed C reached root‐free hyphal compartments via AMF within 1 d and became accessible to hyphosphere bacteria within 1 wk, revealing a clear temporal hierarchy of C acquisition among soil microbial groups. This directional C flow was mirrored by a return of ^15^N from the root‐free SC back to plant roots and shoots, completing a tripartite plant–fungal–bacterial exchange under realistic field conditions. The microbial communities mediating these fluxes were shaped by their position along the rhizosphere–hyphosphere gradient, with hyphal‐dominated compartments (i.e. root free) exhibiting lower diversity, stronger community filtering, and proportionally more positive fungal–bacterial associations.

### Reciprocal carbon–nitrogen exchange and timeframes of plant‐fixed C allocation to soil microbial groups in the (mycor)rhizosphere and hyphosphere

Soil hyphal networks potentially facilitate coupling between plant C inputs and microbial nutrient mobilization. In agreement, the observed patterns of ^13^C and ^15^N signatures in soil, plant above‐ and belowground biomass, and microbial biomarkers provide strong evidence for reciprocal C and N transfers.

Our NanoSIMS analyses provide microscale evidence for a rapid incorporation of recently assimilated C into root tissues, consistent with our isotopic measurements of root biomass. From there, plant‐derived C may enter soil microbial pools through multiple pathways, including direct transfer to AMF, uptake from root exudates, dead root cells or biomass in the rhizosphere, or via the turnover, decay, or grazing of living or dead AMF hyphae (Jansa *et al*., 2013). Numerous studies have demonstrated rapid transfer of plant C to AMF within 24 h (Johnson *et al*., 2002; Leake *et al*., 2006; Kaiser *et al*., 2015; Tomè *et al*., 2015), consistent with our observation that AMF constituted one of the earliest and strongest sinks of plant‐fixed C among all microbial groups: the stable abundance and high ^13^C enrichment of the AMF biomarker NLFA 16:1ω5 across all hyphobox compartments, compared with other microbial groups, underscores the dominant role of AMF in belowground C transfer. These rapid transfer dynamics support the role of AMF as efficient channels for newly assimilated photosynthate into soil food webs and as potential contributors to the formation of stable OM pools, including mineral‐associated OM, as reported previously (Jeewani *et al*., 2021; Kakouridis *et al*., 2024).

Despite their similarly rapid incorporation of plant‐derived C, AMF and saprotrophic fungi differed markedly in the spatial extent of ^13^C reallocation within their hyphal networks. AMF received C directly from the plants in root‐near areas and subsequently translocated it to the more distant root‐free SC and across the 4‐mm air gap. Comparable long‐distance C transport by AMF has been reported previously, including transfer across a 3‐mm air gap into a separate soil compartment over a relatively short timescale of 6 wk within a growth chamber (Kakouridis *et al*., 2024). By contrast, saprotrophic fungi primarily incorporated plant‐derived C, likely freshly released rhizodeposits, in zones closer to roots. Supporting this spatial constraint, our previous hyphobox study demonstrated that ^13^C from labeled OM was incorporated by saprotrophic fungi mostly locally (Brandt *et al*., 2025). Together, these findings suggest that while saprotrophic fungi rapidly exploit localized C inputs, AMF uniquely facilitate longer‐distance C–nutrient exchange between plants and spatially separated soil microhabitats under realistic field conditions in mature and diverse grassland ecosystems.

In contrast to fungi, bacterial groups showed only minimal ^13^C incorporation and only after 7 d, indicating strongly delayed access to recent photosynthate. This aligns with evidence that saprotrophic fungi are more efficient than bacteria at acquiring rhizodeposition‐derived C (Huang *et al*., 2020) and reinforces the view that fungi are key regulators of rhizosphere C flows (Ballhausen & de Boer, 2016). Although Gram‐negative bacteria in particular are often considered competitive consumers of rhizodeposits (Vandenkoornhuyse *et al*., 2007), bacterial groups in root‐proximal zones showed no detectable ^13^C incorporation, whereas a slight increase in bacterial PLFA enrichment in the SC by Day 7 suggests that bacteria begin to access plant‐derived C indirectly at later stages in root‐free, hyphae‐dominated zones. Such delayed, likely fungal‐mediated bacterial uptake is consistent with earlier studies showing that bacterial incorporation of plant‐derived C in the hyphosphere occurs on longer timescales than fungal uptake (Denef *et al*., 2007; Kakouridis *et al*., 2024). Similarly, Drigo *et al*. (2010) reported rapid C transfer from plants to AM hyphae followed by subsequent transfer to Gram‐negative bacteria after 4–5 d in a pot experiment, a pattern now corroborated under field conditions. Because only AMF, and not saprotrophic fungi, showed ^13^C enrichment in the SC, we infer that bacteria there accessed plant‐derived C primarily via AM fungal exudates in the hyphosphere, which may in turn promote their recruitment for nutrient cycling, including N mobilization from the added urea. In line with this, the close spatial association between hyphae‐like structures and ^15^N‐enriched microsites in the SC, as indicated by NanoSIMS, supports the involvement of fungi, and possibly hyphosphere‐associated bacteria, in urea processing. Owing to the low labeling intensity, however, we could not identify the specific bacterial taxa incorporating ^13^C, and their functional roles warrant further work.

Acting as central intermediaries, AMF channel photosynthates toward N hotspots and return mobilized N to plants, bridging the spatial gap between roots and distant nutrient sources. Consistent with this, AMF have been shown to transport litter‐derived N to their host plant, while altering C flow through, and the composition of, the surrounding soil microbial community (Herman *et al*., 2012; Nuccio *et al*., 2013). Alongside AMF‐mediated pathways, we cannot exclude that saprotrophic fungi also contributed to the observed ^15^N transfer to plants, for example by assimilating added urea‐N and subsequently releasing it in root‐proximal zones through hyphal turnover or exudation. Quantitatively, this fungal‐mediated N return was substantial from a plant perspective: 16.9% of the ^15^N initially supplied to the root‐free SC was recovered in plant biomass after 3 months. This demonstrates that hyphal networks did not merely redistribute N within the soil but delivered it into actively growing plant tissues across a spatial gap that roots could not bridge directly, highlighting the relevance of these belowground processes for plant N acquisition in structurally heterogeneous grassland soils. Consistent with the experimental context, 17.3% of the added ^15^N remained in the SC after 3 months: the hyphobox compartments were filled with sterilized soil, so microbial colonization and N turnover had to re‐establish from the surrounding soil, as reflected in their low PLFA contents. Plants were also not dependent on the added urea for N, since their roots simultaneously accessed the native and residual fertilizer N of the surrounding soil.

Altogether, our findings particularly identify AMF as primary early ‘gatekeepers’ of freshly fixed plant C in root‐distant soil microhabitats. Bacterial groups benefit more gradually, likely via fungal‐mediated pathways and secondary consumption processes associated with nutrient mobilization in the hyphosphere. Ultimately, these fungal‐mediated fluxes reconnect the mobilized nutrients with the plant, securing host N acquisition from root‐distant soil zones that roots cannot reach directly.

### Fungal and bacterial communities across root‐ and hyphal‐dominated soil microhabitats

The observed C and N fluxes described above were embedded in, and likely partly shaped by, the microbial communities that assembled within each microhabitat. We found that the distinct experimentally separated soil niches developed distinct fungal and bacterial community compositions after 3 months of assembly under field conditions. We further observed a pronounced decline in microbial diversity from the BS toward the inner, root‐free compartments. In line with these patterns, cross‐kingdom network analyses revealed a consistent decline in the number of hubs, nodes, and edges and a lower average node degree, alongside an increase in the number and connectivity of modules, indicating an increasing organization of compartments.

Saprotrophic fungi were abundant across hyphobox compartments, consistent with their ability to exploit a wide range of organic substrates, and dominated the fungal contribution to co‐occurrence networks, forming positive and negative associations with a wide range of bacterial phyla. By contrast, AMF (i.e. *Claroideoglomus*) occurred in the reconstructed fungal–bacterial networks only in the RC, where they were associated with specific bacteria. The bacterial groups positively linked to AMF in our experiment largely correspond to phyla repeatedly reported as abundant or enriched in AMF‐associated hyphosphere communities (Zhang *et al*., 2022). For instance, *Pseudomonadota* and *Bacillota* were identified as key components of hyphosphere microbial networks associated with *Rhizophagus irregularis* (Faghihinia *et al*., 2024). Similarly, *Pseudomonadota*, *Acidobacteriota*, *Bacillota*, and *Verrucomicrobiota* were reported among the most abundant hyphospheric bacteria (Duan *et al*., 2024), supporting the idea that these phyla constitute recurring members of a hyphosphere‐associated microbiome. By contrast, despite *Fibrobacterales* being proposed as core hyphosphere members in different studies (Emmett *et al*., 2021; Wang *et al*., 2023), their consistently negative association with *Claroideoglomus* suggests that relationships between AMF and putative core hyphosphere bacteria can be species‐ or context‐dependent. Finding AMF only in the RC network might support the notion that AMF–bacterial associations are particularly prominent near roots, where plant C supply and microbial activity peak. This contrasts with Yuan *et al*. (2021), who reported fewer AMF–bacterial associations in the rhizosphere and more in root‐distant BS containing root litter. At the same time, AMF occurred at comparably low relative abundance in our sequencing data despite their clear functional relevance indicated by lipid biomarkers and isotope tracing. This discrepancy likely reflects methodological limitations of amplicon‐based approaches for AMF and an important limitation of association‐based network analyses, which may underrepresent functionally important but low‐abundance taxa (Faust, 2021). The absence of AMF from BS, HC, and SC networks therefore probably results from the use of nonspecific ITS primers and stringent prenetwork filtering, rather than from a lack of ecological relevance.

While network size (number of nodes and edges) generally decreased from root‐ toward hyphal‐dominated compartments, the proportion of positive fungal–bacterial associations increased. Importantly, positive and negative associations in co‐occurrence networks reflect statistical patterns of co‐occurrence and mutual exclusion, respectively, and do not directly indicate biotic interactions between taxa, or their nature. Such patterns can arise from shared environmental preferences, similar resource requirements, dispersal dynamics, or biotic interactions, and disentangling these drivers remains a fundamental challenge in microbial network analysis (D'Amen *et al*., 2018; Jiang *et al*., 2021; Lee *et al*., 2022). Moreover, the increased proportion of positive associations in the HC and SC could partly reflect the reduced community complexity in these compartments, where the smaller number of taxa may affect the detectability of association patterns. With these caveats in mind, several nonexclusive mechanisms may have contributed to the observed shift. First, the air gap introduced in the ingrowth cores represented a substantial dispersal barrier for bacteria that was likely overcome with the help of fungal hyphae (Ingham *et al*., 2011; Mafla‐Endara *et al*., 2021), potentially selecting for taxa that depend on hyphal‐mediated dispersal and thus co‐occur with fungi. Second, fungal–bacterial co‐occurrence is strongly modulated by resource availability (Zheng *et al*., 2018; Yuan *et al*., 2021; Lee *et al*., 2022; Faghihinia *et al*., 2024), and accordingly, we previously showed that hyphosphere community assembly is driven by OM quality in an earlier study utilizing hyphoboxes (Brandt *et al*., 2025). Fungal exudates and necromass represent key resources that can support bacteria in the hyphosphere (Kohlmeier *et al*., 2005; Toljander *et al*., 2006, 2007; Lecomte *et al*., 2011). In the SC specifically, this potential fungal C supply was reflected in the rapid AMF‐mediated ^13^C enrichment documented above, which, together with the added root litter and urea, may have reduced competition and favored co‐occurring taxa that depend on hyphal‐derived C, although our data cannot confirm whether these patterns represent truly facilitative interactions. Conversely, the higher frequency of negative associations in the BS and RC may reflect stronger competition among a larger and more diverse microbial community.

Overall, our findings suggest that, while rhizosphere environments supported more diverse microbial assemblages and complex interaction networks, hyphae‐dominated (i.e. root free) zones exhibited reduced diversity and simplified network structures. However, because the inner compartments were located at increasing distances from the surrounding intact soil serving as the primary source of microbial colonization, the relative contributions of ecological filtering and dispersal limitation to these patterns cannot be fully disentangled in our design. Future studies using modified ingrowth container designs, for example with hyphal compartments positioned directly adjacent to soil or with compartments inoculated with a microbial wash, would help to resolve this question. Accordingly, differences between BS and the hyphobox compartments should be interpreted cautiously, as they reflect the contrast between native unsterilized field soil and sterilized, recolonized hyphobox soil, whereas the RC–HC–SC comparisons more directly represent the experimentally imposed root‐to‐hyphae microhabitat gradient.

### Conclusions

Combining compartmented ingrowth cores with isotope tracing under field conditions, we found that soil hyphal networks, and AMF in particular, occupied a central role in the reciprocal plant–soil C–N exchange. AMF acted as dominant initial sinks for plant‐fixed C and enabled its transfer across several centimeters, including a 4‐mm air gap, to nutrient‐rich microhabitats beyond the rhizosphere. In these root‐free zones, hyphosphere bacteria accessed recent photosynthate only subsequently, likely primarily via fungal‐derived C pools. In turn, a substantial share of the ^15^N supplied to the root‐distant SC was returned to plant shoots and roots, demonstrating that hyphal networks secure plant nutrient acquisition from soil zones that roots cannot reach directly. These C and N fluxes were embedded in microbial communities that shifted along the rhizosphere–hyphosphere gradient toward lower diversity as well as simplified co‐occurrence networks with an increased prevalence of positive fungal–bacterial associations. Such patterns may reflect fungal exudation supporting bacterial growth, hyphal‐mediated dispersal of bacteria across the air gap, or bacterial nutrient mobilization benefiting fungi, but could equally arise from shared environmental preferences or reduced competition at low microbial densities. Future studies are needed to clarify whether these patterns reflect true facilitative interactions. Importantly, by extending insights from controlled laboratory experiments to a more complex and realistic grassland system, this study links plant C allocation to soil microbial functioning and underscores the need to explicitly consider fungal‐mediated processes in plant nutrient acquisition in grassland ecosystems.

## Competing interests

None declared.

## Author contributions

EK, SM, AA and LB planned and designed the research. AA and LB conducted the fieldwork. LB, AA, A‐CW, SSchweizer, SSchulz, SM and EK contributed to the data analysis, collection, and interpretation. LB wrote the first draft of the manuscript. All authors contributed to the final manuscript.

## Disclaimer

The New Phytologist Foundation remains neutral with regard to jurisdictional claims in maps and in any institutional affiliations.

## Supporting information


**Fig. S1** Construction of the hyphobox and its compartments.
**Fig. S2** Layout of hyphobox burial at the grassland site.
**Fig. S3** CO_2_ concentration monitoring during pre‐experimental plant CO_2_ consumption tests and ^13^CO_2_ pulse labeling.
**Fig. S4** Individual secondary ion images from NanoSIMS analysis of the four soil regions shown in the main text (Fig. 4).
**Fig. S5** Average NanoSIMS secondary ion signal intensities across structural segments.
**Fig. S6** Plant shoot and root biomass at the time of sampling.
**Fig. S7** ^13^C and ^15^N atom‐% in plant shoots, roots, and soils across bulk soil and hyphobox compartments.
**Fig. S8** Soil water content across bulk soil and hyphobox compartments.
**Fig. S9** Variations in the content of the neutral lipid fatty acid 16:1ω5 between labeling treatments and sampling days.
**Fig. S10** NanoSIMS ion images of ^27^Al^16^O^−^ and ^31^P^−^ in the imaged regions.
**Fig. S11** Diversity indices of bacterial and fungal communities.
**Fig. S12** Nonmetric multidimensional scaling ordination of bacterial and fungal communities.
**Fig. S13** Differential abundance of bacterial phyla across compartments.
**Fig. S14** Differential abundance of fungal phyla across compartments.
**Table S1** Analyses performed for each individual hyphobox.
**Table S2** Number of reads retained per sample at each step of the DADA2 pipeline for 16S rRNA gene amplicon sequencing.
**Table S3** Number of reads retained per sample at each step of the DADA2 pipeline for ITS amplicon sequencing.
**Table S4** Summary of linear and linear mixed‐effects models after model simplification testing effects of Compartment, Time and Treatment on different variables.
**Table S5** Pairwise comparisons of the labeled vs unlabeled treatments within each time × compartment combination for ^13^C incorporation into microbial fatty acids.
Please note: Wiley is not responsible for the content or functionality of any Supporting Information supplied by the authors. Any queries (other than missing material) should be directed to the *New Phytologist* Central Office.

## Data Availability

Raw amplicon sequences are deposited at the NCBI Sequence Read Archive under the BioProject ID PRJNA1415267. All other data are available in the Biodiversity Exploratories information system (BExIS) under the dataset ID 32469 (Brandt, 2026; https://doi.org/10.71615/bexis.32469).
