# Supplementary material for: Reciprocal transport of carbon and nitrogen between plants and the hyphosphere microbiome in a temperate grassland
Source: New Phytol. 2026 Sep 10;252(4):1602–19. doi: 10.1111/nph.71576 (PMC13669818; doi:10.1111/nph.71576)
Supplement: Supplementary file 1 [file NPH-252-1602-s001.docx]

## *New Phytologist* Supporting Information

Article title: **Reciprocal transport of carbon and nitrogen between plants and the hyphosphere microbiome in a temperate grassland**

Authors: Luise Brandt, Anna Abrahão, Ann-Christin Wicht, Stefanie Schulz, Michael Schloter, Steffen Schweizer, Sven Marhan, Ellen Kandeler

Article acceptance date: 27 August 2026

The following Supporting Information is available for this article:

Figures

**Fig. S1:** Construction of the hyphobox and its compartments.

**Fig. S2:** Layout of hyphobox burial at the grassland site.

**Fig. S3:** CO₂ concentration monitoring during pre-experimental plant CO₂ consumption tests and ¹³CO₂ pulse labeling.

**Fig. S4:** Individual secondary ion images from NanoSIMS analysis of the four soil regions shown in the main text (Fig. 4).

**Fig. S5:** Average NanoSIMS secondary ion signal intensities across structural segments.

**Fig. S6:** Plant shoot and root biomass at the time of sampling.

**Fig. S7**: ^13^C and ^15^N atom-% in plant shoots, roots, and soils across bulk soil and hyphobox compartments.

**Fig. S8:** Soil water content across bulk soil and hyphobox compartments.

**Fig. S9:** Variations in the content of the neutral lipid fatty acid (NLFA) 16:1ω5 between labeling treatments and sampling days.

**Fig. S10:** NanoSIMS ion images of ²⁷Al¹⁶O⁻ and ³¹P⁻ in the imaged regions.

**Fig. S11:** Diversity indices of bacterial and fungal communities.

**Fig. S12:** Non-metric multidimensional scaling (NMDS) ordination of bacterial and fungal communities.

**Fig. S13:** Differential abundance of bacterial phyla across compartments.

**Fig. S14:** Differential abundance of fungal phyla across compartments.

Tables

**Table S1:** Analyses performed for each individual hyphobox.

**Table S2:** Number of reads retained per sample at each step of the DADA2 pipeline for 16S rRNA gene amplicon sequencing.

**Table S3:** Number of reads retained per sample at each step of the DADA2 pipeline for ITS amplicon sequencing.

**Table S4:** Summary of linear (LM) and linear mixed-effects models (LMM) after model simplification testing effects of Compartment, Time and Treatment on different variables.

**Table S5:** Pairwise comparisons of the labeled vs. unlabeled treatments within each Time × Compartment combination for ^13^C incorporation into microbial fatty acids.

**Fig. S1:** **Construction of the hyphobox and its compartments.** (a) Empty polyvinyl chloride (PVC) tube used to contain five compartments, arranged in the following order: (mycor)rhizosphere compartment (RC), hyphosphere compartment (HC), substrate compartment (SC), HC, and RC. Air gaps of 4 mm were installed between the SC and HCs using additional plastic rings (4 mm height). Compartments were secured with screws. (b) Individual components of a single compartment: one central plastic ring (height 1 cm, outer diameter 7.5 cm, inner diameter 7 cm), two meshes (diameter 9 cm), and two outer plastic rings (height 4 mm, inner diameter 7.5 cm, outer diameter 9 cm) used to fix the meshes to the central ring. (c) Empty assembled compartment with 500-µm meshes, used as a RC. (d) Empty assembled compartment with 50-µm meshes, used as either a HC or SC. (e) Collection of a small soil core from a RC for NanoSIMS analysis.

**Fig. S2:** **Layout of hyphobox burial at the grassland site.** Thirty-six hyphoboxes were buried in a fully randomized layout on an 8 × 5 m area, with 1-m spacing between hyphobox positions. Each square represents the position of a labeling chamber beneath which an individual hyphobox had been installed three months prior to pulse labeling. Numbers above squares indicate the unique hyphobox IDs (Supporting Information Tab. S1 informs about analyses performed for each individual hyphobox). Box colours denote the sampling time following the ^13^CO_2_-pulse labelling (1, 3, or 7 days). For each hyphobox, treatments are depicted (^15^N-labelled and/or ^13^C-labelled). Hyphobox replication was n = 11 for the ^15^N-^13^C-labelled, n = 13 for the ^13^C-only-labelled, and n = 12 for the unlabelled treatment.

**Fig. S3:** **CO₂ concentration monitoring during pre-experimental plant CO₂ consumption tests and ¹³CO₂ pulse labeling.** (a) CO₂ concentration in closed chambers over time during plant CO₂ consumption tests conducted on June 2, 2021 (n = 7 measurements). These tests were used to estimate plant CO₂ uptake rates and to calibrate the required amount of NaHCO₃ for the pulse labeling. (b) CO₂ concentration monitored in the unlabeled control chamber (receiving ¹²CO₂) during the three consecutive labeling days (July 19–21, 2021). CO₂ was maintained within the target range of 380–800 ppm by stepwise addition of H₃PO₄ to NaHCO₃, with fluctuations reflecting acid additions and plant CO₂ uptake.

**Fig. S4:** **Individual secondary ion images from NanoSIMS analysis of the four soil regions shown in the main text (Fig. 4).** Rows show the distribution of the measured ion species: ^16^O^-^, ^12^C_2_^-^, ^12^C^13^C^-^, ^12^C^14^N^-^, ^12^C^15^N^-^, ^31^P^-^, and ^27^Al^16^O^-^; each column corresponds to one measured region. Ion signals are displayed as counts, with intensity increasing from blue (low) to red (high); scales are adjusted per image for optimal contrast and are therefore not directly comparable between panels.

**Fig. S5:** **Average NanoSIMS secondary ion signal intensities across structural segments.** Mean ion counts per pixel for ^16^O^-^, ^12^C_2_^-^, ^12^C^13^C^-^, ^12^C^14^N^-^, ^12^C^15^N^-^, ^31^P^-^, and ^27^Al^16^O^-^ within non-enriched and isotopically enriched regions of resin, mineral, and organic matter (OM) segments, averaged across the four regions shown in Fig. 4a–d. Note the break in the y-axis to accommodate the wide range in signal intensities across ion species.

**Fig. S6:** **Plant shoot and root biomass at the time of sampling.** (a) plant shoot biomass in a 40x40 area covered by the labelling chamber and (b) root biomass in the rhizosphere compartment at the different sampling days, summarized across labeling treatments. Each mean represents n=12 observations. Dots represent means and bars standard deviations. Letters denote significant (*p* < 0.05) differences between timepoints according to linear modeling.

**Fig. S7:** **^13^C and ^15^N atom-% in plant shoots, roots, and soils across bulk soil and hyphobox compartments.** (a–c) ^13^C atom-% and (d–f) ^15^N atom-% in (a, d) plant shoot biomass harvested from a 40 cm × 40 cm area covered by the labeling chamber, (b, e) root biomass of the (mycor)rhizosphere compartment (RC), and (c, f) the bulk soil (BS) and soils of the different hyphobox compartments, i.e. the RC, hyphosphere compartment (HC), and substrate compartment (SC), at one, three, and seven days after ^13^CO_2_ pulse-labeling. ^13^C atom-% values are pooled across the ^13^C-only- and ^13^C-^15^N-labeled treatments (n = 8 per timepoint, except roots at day three where n = 7); ^15^N atom-% values represent the ^13^C-^15^N-labeled treatment (n = 4 per timepoint, except day 3 where n = 3). Dotted lines indicate the mean of the unlabeled control treatment, averaged across sampling days (n = 12). Dots show means and bars standard deviations.

**Fig. S8:** **Soil water content across bulk soil and hyphobox compartments.** Shown are means (dots) and standard deviations (bars) of soil water contents at the time of sampling, averaged across treatments and sampling days, for the bulk soil (BS), (mycor)rhizosphere compartment (RC), hyphosphere compartment (HC), and substrate compartment (SC). Each mean represents n = 36 observations. Different letters indicate significant differences among compartments (*p* < 0.05) based on linear mixed-effects modeling.

**Fig. S9:** **Variations in the content of the neutral lipid fatty acid (NLFA) 16:1ω5 between labeling treatments and sampling days.** Shown are results for the bulk soil (BS), (mycor)rhizosphere compartment (RC), hyphosphere compartment (HC), and substrate compartment (SC). Dots represent means and bars standard deviations.

**Fig. S10:** **NanoSIMS ion images of ²⁷Al¹⁶O⁻ and ³¹P⁻ in the imaged regions.** RGB overlay images showing the distribution of ²⁷Al¹⁶O⁻ (magenta) and ³¹P⁻ (green) in (a) the bulk soil, (b) the (mycor)rhizosphere compartment, (c) the hyphosphere compartment, and (d) the substrate compartment, corresponding to the same regions shown in Fig. 4a–d in the main text.

**Fig. S11:** **Diversity indices of bacterial and fungal communities.** Panels show bacterial a) Shannon diversity and b) Simpson diversity, as well as fungal c) Shannon diversity and d) Simpson diversity. Data points are coloured by their assignment either to the bulk soil (BS), or one of the hyphobox compartments, i.e., the rhizosphere compartment (RC), hyphosphere compartment (HC), or substrate compartment (SC). Each mean represents n = 8 observations. Dots represent means and bars standard deviations. Letters denote significant (*p* < 0.05) differences between compartments according to linear mixed-effects modeling.

**Fig. S12:** **Non-metric multidimensional scaling (NMDS) ordination of bacterial and fungal communities.** a) bacterial (stress value: 0.14) and b) fungal community compositions (stress value: 0.15) across compartments (bulk soil, BC; (mycor)rhizosphere compartment, RC; hyphosphere compartment, HC; substrate compartment, SC) based on Bray–Curtis dissimilarities calculated from relative abundance data. Each point represents one sample, and colors indicate compartments. Distances between points reflect differences in community composition, with closer points indicating more similar communities. For bacteria, BS samples were omitted from the plot to improve visualization of differences among the other compartments, as their pronounced compositional divergence dominated the ordination space.

**Fig. S13:** **Differential abundance of bacterial phyla across compartments.** Forest plots showing log₂ fold changes (LFCs) (±95% confidence intervals) for the 15 most abundant bacterial phyla across the three contrasts: rhizosphere compartment (RC) vs bulk soil (BS), hyphosphere compartment (HC) vs RC, and substrate compartment (SC) vs HC. Phyla with *p* < 0.05 and |LFC| > 1.5 are highlighted in black, while all others are shown in grey. Positive values indicate relative enrichment, and negative values indicate depletion.

**Fig. S14:** **Differential abundance of fungal phyla across compartments.** Forest plots showing log₂ fold changes (LFCs) (±95% confidence intervals) for the 15 most abundant fungal phyla across the three contrasts: rhizosphere compartment (RC) vs bulk soil (BS), hyphosphere compartment (HC) vs RC, and substrate compartment (SC) vs HC. Phyla with *p* < 0.05 and |LFC| > 1.5 are highlighted in black, while all others are shown in grey. Positive values indicate relative enrichment, and negative values indicate depletion.

**Table S1: Analyses performed for each individual hyphobox.** Overview of each hyphobox, including the exact dates of ^13^CO₂ pulse labeling and sampling, and all performed analyses. Abbreviations: C, carbon; N, nitrogen; PLFA, phospholipid fatty acid; NLFA, neutral lipid fatty acid; NanoSIMS, nanoscale secondary ion mass spectrometry. Different blue background colors denote timepoints, while ^13^C and ^15^N labeling treatments are highlighted in green (labeled) and red (unlabeled).

| Hyphobox  ID | ^13^C Labeling date (D/M/Y) | Sampling date  (D/M/Y) | Days | ^15^N | ^13^C | C, N | PLFAs  /NLFA | Amplicon  sequencing | NanoSIMS |
| --- | --- | --- | --- | --- | --- | --- | --- | --- | --- |
| 1 | 21.07.2021 | 22.07.2021 | 1 | yes | yes | x |  |  |  |
| 7 | 21.07.2021 | 22.07.2021 | 1 | yes | yes | x |  |  | x |
| 8 | 21.07.2021 | 22.07.2021 | 1 | yes | yes | x |  |  |  |
| 11 | 21.07.2021 | 22.07.2021 | 1 | yes | yes | x |  |  |  |
| 13 | 21.07.2021 | 22.07.2021 | 1 | no | yes | x | x | x |  |
| 16 | 21.07.2021 | 22.07.2021 | 1 | no | yes | x | x | x |  |
| 17 | 21.07.2021 | 22.07.2021 | 1 | no | yes | x | x | x |  |
| 21 | 21.07.2021 | 22.07.2021 | 1 | no | yes | x | x | x |  |
| 27 | - | 22.07.2021 | 1 | no | no | x | x | x |  |
| 29 | - | 22.07.2021 | 1 | no | no | x | x | x |  |
| 34 | - | 22.07.2021 | 1 | no | no | x | x | x | x |
| 36 | - | 22.07.2021 | 1 | no | no | x | x | x |  |
| 37 | 20.07.2021 | 23.07.2021 | 3 | no | yes | x |  |  |  |
| 9 | 20.07.2021 | 23.07.2021 | 3 | yes | yes | x |  |  |  |
| 10 | 20.07.2021 | 23.07.2021 | 3 | yes | yes | x |  |  |  |
| 12 | 20.07.2021 | 23.07.2021 | 3 | yes | yes | x |  |  |  |
| 15 | 20.07.2021 | 23.07.2021 | 3 | no | yes | x | x |  |  |
| 18 | 20.07.2021 | 23.07.2021 | 3 | no | yes | x | x |  |  |
| 20 | 20.07.2021 | 23.07.2021 | 3 | no | yes | x | x |  |  |
| 23 | 20.07.2021 | 23.07.2021 | 3 | no | yes | x | x |  |  |
| 28 | - | 23.07.2021 | 3 | no | no | x | x |  |  |
| 30 | - | 23.07.2021 | 3 | no | no | x | x |  |  |
| 31 | - | 23.07.2021 | 3 | no | no | x | x |  |  |
| 35 | - | 23.07.2021 | 3 | no | no | x | x |  |  |
| 2 | 19.07.2021 | 26.07.2021 | 7 | yes | yes | x |  |  |  |
| 4 | 19.07.2021 | 26.07.2021 | 7 | yes | yes | x |  |  |  |
| 5 | 19.07.2021 | 26.07.2021 | 7 | yes | yes | x |  |  |  |
| 6 | 19.07.2021 | 26.07.2021 | 7 | yes | yes | x |  |  | x |
| 14 | 19.07.2021 | 26.07.2021 | 7 | no | yes | x | x |  |  |
| 19 | 19.07.2021 | 26.07.2021 | 7 | no | yes | x | x |  |  |
| 22 | 19.07.2021 | 26.07.2021 | 7 | no | yes | x | x |  |  |
| 24 | 19.07.2021 | 26.07.2021 | 7 | no | yes | x | x |  |  |
| 25 | - | 26.07.2021 | 7 | no | no | x | x |  |  |
| 26 | - | 26.07.2021 | 7 | no | no | x | x |  |  |
| 32 | - | 26.07.2021 | 7 | no | no | x | x |  |  |
| 33 | - | 26.07.2021 | 7 | no | no | x | x |  | x |

**Table S2: Number of reads retained per sample at each step of the DADA2 pipeline for 16S rRNA gene amplicon sequencing.**

| Hyphobox ID | Compartment | input | filtered | denoisedF | denoisedR | merged | nonchim |
| --- | --- | --- | --- | --- | --- | --- | --- |
| 13 | BS | 77382 | 57388 | 54429 | 54707 | 45939 | 45716 |
| 16 | BS | 80169 | 61370 | 58267 | 58210 | 48115 | 47884 |
| 17 | BS | 79909 | 60792 | 57674 | 57720 | 46896 | 46687 |
| 21 | BS | 91400 | 69694 | 66189 | 66466 | 54887 | 54707 |
| 27 | BS | 93229 | 71007 | 67444 | 67717 | 56960 | 56733 |
| 29 | BS | 118383 | 87435 | 83881 | 84035 | 72718 | 72373 |
| 34 | BS | 89032 | 68828 | 65426 | 65674 | 54038 | 53794 |
| 36 | BS | 143224 | 112000 | 107223 | 107077 | 90684 | 90284 |
| 13 | RC | 37686 | 26829 | 24854 | 25189 | 21327 | 21252 |
| 16 | RC | 80662 | 62384 | 59561 | 59760 | 52695 | 52376 |
| 17 | RC | 71173 | 54756 | 52510 | 52503 | 46917 | 46713 |
| 21 | RC | 80876 | 64120 | 61280 | 61449 | 53664 | 52987 |
| 27 | RC | 94278 | 73504 | 70537 | 70481 | 62322 | 61846 |
| 29 | RC | 102615 | 73041 | 70322 | 70600 | 63505 | 62978 |
| 34 | RC | 116462 | 85504 | 81872 | 82112 | 72595 | 71915 |
| 36 | RC | 121718 | 94902 | 91472 | 91301 | 80127 | 79650 |
| 13 | HC | 96182 | 67933 | 66062 | 66459 | 61072 | 60272 |
| 16 | HC | 113345 | 90489 | 88275 | 88475 | 82416 | 80849 |
| 17 | HC | 91797 | 71602 | 69905 | 69974 | 65320 | 64570 |
| 21 | HC | 104310 | 76622 | 74814 | 74985 | 70006 | 69037 |
| 27 | HC | 108058 | 83706 | 81931 | 81941 | 75659 | 74711 |
| 29 | HC | 130650 | 104010 | 102307 | 102490 | 96429 | 95079 |
| 34 | HC | 93877 | 72684 | 70615 | 70723 | 64314 | 63399 |
| 36 | HC | 135271 | 104409 | 102536 | 102599 | 96536 | 95165 |
| 13 | SC | 87366 | 67386 | 65825 | 66052 | 61205 | 59311 |
| 16 | SC | 181150 | 143625 | 141451 | 141838 | 134631 | 131182 |
| 17 | SC | 112033 | 75897 | 74457 | 74384 | 70342 | 69899 |
| 21 | SC | 79386 | 62295 | 60879 | 60980 | 57091 | 56735 |
| 27 | SC | 74506 | 57728 | 56548 | 56531 | 52650 | 52081 |
| 29 | SC | 95133 | 74979 | 74039 | 74091 | 70445 | 69196 |
| 34 | SC | 523666 | 398534 | 395520 | 396180 | 380130 | 366893 |
| 36 | SC | 96876 | 76820 | 75562 | 75815 | 71128 | 69776 |
| negative control | | 47 | 20 | 2 | 2 | 0 | 0 |
| negative control | | 39 | 10 | 1 | 1 | 0 | 0 |
| negative control | | 32 | 11 | 5 | 5 | 5 | 5 |
| standard soil | | 102054 | 71660 | 66977 | 67008 | 52217 | 52093 |
| standard soil | | 133000 | 101622 | 96190 | 96113 | 76055 | 75757 |
| standard soil | | 116992 | 89442 | 84045 | 84245 | 65778 | 65611 |

**Table S3: Number of reads retained per sample at each step of the DADA2 pipeline for ITS amplicon sequencing.**

| Hyphobox ID | Compartment | input | filtered | denoisedF | denoisedR | merged | nonchim |
| --- | --- | --- | --- | --- | --- | --- | --- |
| 13 | BS | 53055 | 30036 | 39518 | 39277 | 37096 | 34677 |
| 16 | BS | 55760 | 30708 | 38794 | 38907 | 36849 | 35664 |
| 17 | BS | 57429 | 30625 | 40665 | 40710 | 37421 | 36011 |
| 21 | BS | 48157 | 25518 | 34220 | 34075 | 31250 | 30610 |
| 27 | BS | 50295 | 27142 | 34608 | 34722 | 32683 | 32137 |
| 29 | BS | 69690 | 36495 | 49443 | 49492 | 45015 | 43201 |
| 34 | BS | 63427 | 33946 | 45385 | 45471 | 43020 | 42292 |
| 36 | BS | 78270 | 44691 | 58950 | 58829 | 55100 | 52870 |
| 13 | RC | 43937 | 24163 | 32188 | 32026 | 30341 | 30072 |
| 16 | RC | 46925 | 27119 | 34066 | 34124 | 30917 | 29899 |
| 17 | RC | 52974 | 29890 | 37748 | 37853 | 32627 | 31548 |
| 21 | RC | 65080 | 36755 | 48948 | 48874 | 44148 | 41089 |
| 27 | RC | 81405 | 46399 | 60618 | 60504 | 57385 | 54798 |
| 29 | RC | 94567 | 56584 | 73216 | 73145 | 70517 | 66611 |
| 34 | RC | 75276 | 41284 | 52438 | 52356 | 49244 | 46865 |
| 36 | RC | 78440 | 44722 | 57446 | 57050 | 53069 | 49269 |
| 13 | HC | 87616 | 52830 | 67514 | 67304 | 65751 | 63863 |
| 16 | HC | 96190 | 60190 | 75033 | 74975 | 72239 | 69240 |
| 17 | HC | 109518 | 64329 | 82696 | 82599 | 69490 | 64597 |
| 21 | HC | 36851 | 21029 | 26619 | 26657 | 25013 | 23849 |
| 27 | HC | 40387 | 25635 | 31193 | 31096 | 29959 | 28405 |
| 29 | HC | 42918 | 28550 | 34266 | 34236 | 33780 | 32295 |
| 34 | HC | 40556 | 25178 | 30990 | 30948 | 29947 | 29063 |
| 36 | HC | 32956 | 19768 | 24048 | 23868 | 22723 | 22165 |
| 13 | SC | 41039 | 23411 | 31345 | 31288 | 30575 | 29889 |
| 16 | SC | 24238 | 14494 | 17785 | 17824 | 17609 | 17466 |
| 17 | SC | 48597 | 31110 | 38193 | 38175 | 35202 | 32652 |
| 21 | SC | 46025 | 25827 | 31751 | 31798 | 27393 | 27383 |
| 27 | SC | 52846 | 31732 | 40184 | 40215 | 38139 | 36841 |
| 29 | SC | 75011 | 44303 | 57730 | 57730 | 55751 | 53052 |
| 34 | SC | 51329 | 31564 | 39584 | 39557 | 38631 | 37739 |
| 36 | SC | 53714 | 30892 | 39179 | 39169 | 35260 | 35231 |
| negative control | | 54 | 14 | 12 | 10 | 10 | 10 |
| negative control | | 14 | 1 | 2 | 2 | 0 | 0 |
| negative control | | 18 | 5 | 4 | 4 | 4 | 4 |
| standard soil | | 39100 | 18673 | 27061 | 26915 | 25291 | 24927 |
| standard soil | | 37852 | 16778 | 25521 | 25375 | 23820 | 23569 |
| standard soil | | 41168 | 18632 | 28141 | 27885 | 26432 | 26286 |

**Table S4: Summary of linear (LM) and linear mixed-effects models (LMM) after model simplification testing effects of Compartment, Time and Treatment on different variables.** Non-significant terms were removed during model simplification (backward elimination). Only effects retained in final models are shown. Test statistics are F values for LM and Wald χ² values for LMM. Degrees of freedom (df) are shown for numerator (and denominator for LM).

| Response variable | Model type | Effect | Test statistic | df | *p* value |
| --- | --- | --- | --- | --- | --- |
| Root biomass | LM | Time | F = 14.951 | 2, 33 | < 0.001 |
| Plant biomass | LM | Time | F = 13.745 | 2, 33 | < 0.001 |
| Soil water content | LMM | Compartment | χ^2^ = 144.09 | 3 | < 0.001 |
| Soil excess ^13^C | LMM | Compartment | χ^2^ = 30.128 | 3 | < 0.001 |
| Plant root excess ^13^C | LM | Compartment | F = 2.9968 | 2, 21 | 0.07163 |
| Plant shoot excess ^13^C | LM | no significant effect | – | – | – |
| Soil excess ^15^N | LMM | Compartment | χ^2^ = 688.9 | 3 | < 0.001 |
| Plant root excess ^15^N | LM | no significant effect | – | – | – |
| Plant shoot excess ^15^N | LM | no significant effect | – | – | – |
| NLFA content AMF | LMM | Compartment | χ^2^ = 63.14 | 3 | < 0.001 |
|  |  | Time | χ^2^ = 9.4 | 2 | 0.009 |
|  |  | Treatment | χ^2^ = 1.19 | 1 | 0.274 |
|  |  | Compartment × Time | χ^2^ = 17.83 | 6 | 0.007 |
|  |  | Compartment × Treatment | χ^2^ = 11.2 | 3 | 0.011 |
| PLFA content saprotrophic fungi | LMM | Compartment | χ^2^ = 463.81 | 3 | < 0.001 |
| PLFA content GP bacteria | LMM | Compartment | χ^2^ = 3704.3 | 3 | < 0.001 |
| PLFA content GN bacteria | LMM | Compartment | χ^2^ = 1754.1 | 3 | < 0.001 |
| Bacterial Shannon diversity | LMM | Compartment | χ^2^ = 317.14 | 3 | < 0.001 |
| Bacterial Simpson diversity | LMM | Compartment | χ^2^ = 131.08 | 3 | < 0.001 |
| Fungal Shannon diversity | LMM | Compartment | χ^2^ = 152.29 | 3 | < 0.001 |
| Fungal Simpson diversity | LMM | Compartment | χ^2^ = 26.201 | 3 | < 0.001 |

**Table S5: Pairwise comparisons of the labeled vs. unlabeled treatments within each Time × Compartment combination for ^13^C incorporation into microbial fatty acids.** Pairwise comparisons were calculated using estimated marginal means (emmeans) from linear mixed-effects models with Tukey adjustment. Prior to analysis, four outliers were excluded: for the fungal PLFA C18:2ω6, values from the SC of hyphobox 20 and from the HC of hyphobox 23, both belonging to the ^13^C-only-labeled treatment at day 3; and for Gram-positive and Gram-negative bacteria, values from the SC of hyphobox 31 belonging to the unlabeled treatment at day 3. Degrees of freedom were estimated using the Kenward-Roger method. Only significant (*p* < 0.05) and marginally significant (0.05 ≤ *p* < 0.10) comparisons are shown.

| Response | Time | Compartment | Estimate | SE | df | t | *p* value |
| --- | --- | --- | --- | --- | --- | --- | --- |
| NLFA ^13^C atom-%  arbuscular mycorrhizal fungi | 1 | BS | -0.275 | 0.048 | 38.9 | -5.71 | <0.001 |
|  | 3 | BS | -0.153 | 0.048 | 38.9 | -3.18 | 0.003 |
|  | 7 | BS | -0.087 | 0.048 | 38.9 | -1.80 | 0.080 |
|  | 1 | RC | -0.319 | 0.048 | 38.9 | -6.63 | <0.001 |
|  | 3 | RC | -0.179 | 0.048 | 38.9 | -3.73 | 0.001 |
|  | 7 | RC | -0.100 | 0.048 | 38.9 | -2.09 | 0.044 |
|  | 1 | HC | -0.301 | 0.048 | 38.9 | -6.25 | <0.001 |
|  | 3 | HC | -0.226 | 0.051 | 43.8 | -4.48 | <0.001 |
|  | 7 | HC | -0.129 | 0.048 | 38.9 | -2.69 | 0.011 |
|  | 1 | SC | -0.121 | 0.048 | 38.9 | -2.52 | 0.016 |
|  | 3 | SC | -0.231 | 0.053 | 48.2 | -4.37 | <0.001 |
|  | 7 | SC | -0.095 | 0.048 | 38.9 | -1.98 | 0.055 |
| PLFA ^13^C atom-% saprotrophic fungi | 1 | BS | -0.047 | 0.012 | 58.2 | -3.80 | <0.001 |
|  | 3 | BS | -0.031 | 0.012 | 58.2 | -2.49 | 0.016 |
|  | 7 | BS | -0.034 | 0.012 | 58.2 | -2.77 | 0.008 |
|  | 1 | RC | -0.041 | 0.012 | 58.2 | -3.29 | 0.002 |
|  | 3 | RC | -0.029 | 0.012 | 58.2 | -2.31 | 0.025 |
|  | 7 | RC | -0.047 | 0.012 | 58.2 | -3.77 | <0.001 |
|  | 1 | HC | -0.081 | 0.012 | 58.2 | -6.54 | <0.001 |
|  | 7 | HC | -0.041 | 0.012 | 58.2 | -3.28 | 0.002 |
| PLFA ^13^C atom-% Gram-negative bacteria | 7 | HC | -0.003 | 0.001 | 39.1 | -3.51 | 0.001 |
|  | 7 | SC | -0.002 | 0.001 | 39.1 | -2.08 | 0.044 |
| PLFA ^13^C atom-% Gram-positive bacteria | 7 | SC | -0.003 | 0.001 | 40.8 | -2.58 | 0.014 |
